# Deterministic Bragg Coherent Diffraction Imaging

**DOI:** 10.1038/s41598-017-01164-x

**Published:** 2017-04-25

**Authors:** Konstantin M. Pavlov, Vasily I. Punegov, Kaye S. Morgan, Gerd Schmalz, David M. Paganin

**Affiliations:** 1 School of Science and Technology, University of New England, Armidale, NSW 2351 Australia; 2 School of Physics and Astronomy, Monash University, VIC, 3800 Australia; 3 Komi Research Center, Ural Division, Russian Academy of Sciences, Syktyvkar, 167982 Russian Federation; 4 Syktyvkar State University, Syktyvkar, 167001 Russian Federation; 5 Institute for Advanced Studies and Chair of Biomedical Physics, Technische Universität München, Bayern, 85748 Germany

## Abstract

A deterministic variant of Bragg Coherent Diffraction Imaging is introduced in its kinematical approximation, for X-ray scattering from an imperfect crystal whose imperfections span no more than half of the volume of the crystal. This approach provides a unique analytical reconstruction of the object’s structure factor and displacement fields from the 3D diffracted intensity distribution centred around any particular reciprocal lattice vector. The simple closed-form reconstruction algorithm, which requires only one multiplication and one Fourier transformation, is not restricted by assumptions of smallness of the displacement field. The algorithm performs well in simulations incorporating a variety of conditions, including both realistic levels of noise and departures from ideality in the reference (i.e. imperfection-free) part of the crystal.

## Introduction

Bragg Coherent Diffraction Imaging (BCDI)^1–3^ by crystalline matter allows one to solve classical crystallographic inverse problems of phase retrieval using oversampling of experimental data^4, 5^ and iterative reconstruction algorithms (see e.g., ref. 6 and references therein). BCDI is a Bragg scattering variant of the forward-scattering approach of Coherent Diffractive Imaging (CDI) implemented by Miao *et al*.^7^ in 1999. Both forward-scattering and Bragg scattering techniques require the coherence length of the incident X-ray wave to be larger than the sample size. This condition is achievable at modern X-ray synchrotron sources that are already able to achieve a coherence volume of the order of several cubic micrometres^8^.

BCDI methods have been applied in a large variety of experimental settings. For example, they were successfully employed to investigate dynamic nanoscale processes^9^, to visualize in three dimensions, the entire network of dislocations present within an individual calcite crystal during repeated growth and dissolution cycles^10^, to report an *in situ* three-dimensional mapping of morphology and strain evolutions in a single-crystal nanocube within a high-pressure environment^11^, to understand the surface related properties of shaped nanocrystals^12^, to determine the stacking fault density in highly defective nanowires^13^, to study non-uniform strain relaxation in strained layer nano-objects^14^ and to indicate the presence of surface adsorbates on facetted nanocrystals^15^. This list of applications is indicative but not exhaustive.

In the case of a non-ideal crystal, containing defects of different kinds, the standard statistical approach of X-ray diffractometry allows one to obtain information averaged over ensembles in both the kinematical^16, 17^ or dynamical approaches (see e.g., ref. 18 and references therein). There is a large variety of crystallographic systems for which characterisation requires statistical diffraction theory approaches. These statistically averaging approaches were used, for instance, to characterise nanostructures, in particular, quantum wires^19^ and arrays of quantum dots^20–22^ (see also ref. 23 and references therein). Chaotically distributed spherical clusters^24^ and correlated dislocations^25^ are also still attractive objects of research. Other interesting objects for application of statistical diffraction approaches are crystallographic systems with included nanoclusters^26^, porous structures^27^, crystals having a net of periodic dislocations^28^ or a periodic domain structure^29^. BCDI avoids such an averaging over the illumination volume, demonstrated by standard methods of high resolution X-ray diffractometry (see e.g., ref. 30 and references therein) employing X-ray beams with a small coherence length. The solution of X-ray diffraction inverse problems in these standard diffractometry methods is often based on minimisation of the discrepancy between the simulated and experimental data^30–33^.

Potentially, the resolution obtained by BCDI is restricted only by the wavelength of the radiation used. Therewith, as mentioned in ref. 34 for present day sources with coherent flux of 10^8^–10^9^ photons per second, the minimum size of crystal, which can be investigated by BCDI is about 60 nm in diameter.

There exists a mathematical proof that non-uniqueness for complex-field reconstruction from Fourier modulus data in 2D and higher dimensions is very rare^35^ for functions not having zeros. However, this does not prove that non-linear iterative reconstruction methods (see review in ref. 6) are able to recover that unique solution. As said in ref. 6: “The presence of noise and limited prior knowledge (loose constraints) increases the number of solutions within the noise level and constraints. Confidence that the recovered image is the correct and unique one can be obtained by repeating the phase-retrieval process using several random starts.” However, the convergence to a particular solution does not mean, in general, that this particular solution is the correct solution, the uniqueness of which is discussed in ref. 35, as clearly demonstrated in the Figure in the Conclusions section in ref. 6. In contrast, deterministic reconstruction methods (see e.g., ref. 36; ref. 37 and references therein) are able to recover the unique solution. Such deterministic solutions come at the price of restricting the class of samples that can be reconstructed.

The deterministic reconstruction method, presented in this paper, also does not suffer from another well-known problem of BCDI iterative methods, namely issues with the reconstruction of large phase excursions^38, 39^. This problem prevents quantitative reconstruction of large displacement fields in crystalline samples, which are extremely likely to occur in practice^38^.

This paper provides a deterministic approach to kinematical BCDI, which allows one to stably recover a unique solution for structure factor and displacement fields in an imperfect crystal even in the case of large atomic displacements. Our analysis is restricted to kinematical X-ray scattering from a single imperfect crystal whose imperfections all lie within one particular half of the said crystal. Under this approximation the inverse problem, of reconstructing both the polarisability and the projection of the displacement field along a particular axis, can be performed by weighting the 3D diffracted intensity with a simple function, and then taking the 3D inverse Fourier transform of the resulting product. This remarkably simple reconstruction process directly produces the unique solution to the coherent diffraction imaging inverse problem studied in our paper, subject to the previously stated assumptions.

## Theory

**Figure 1:**
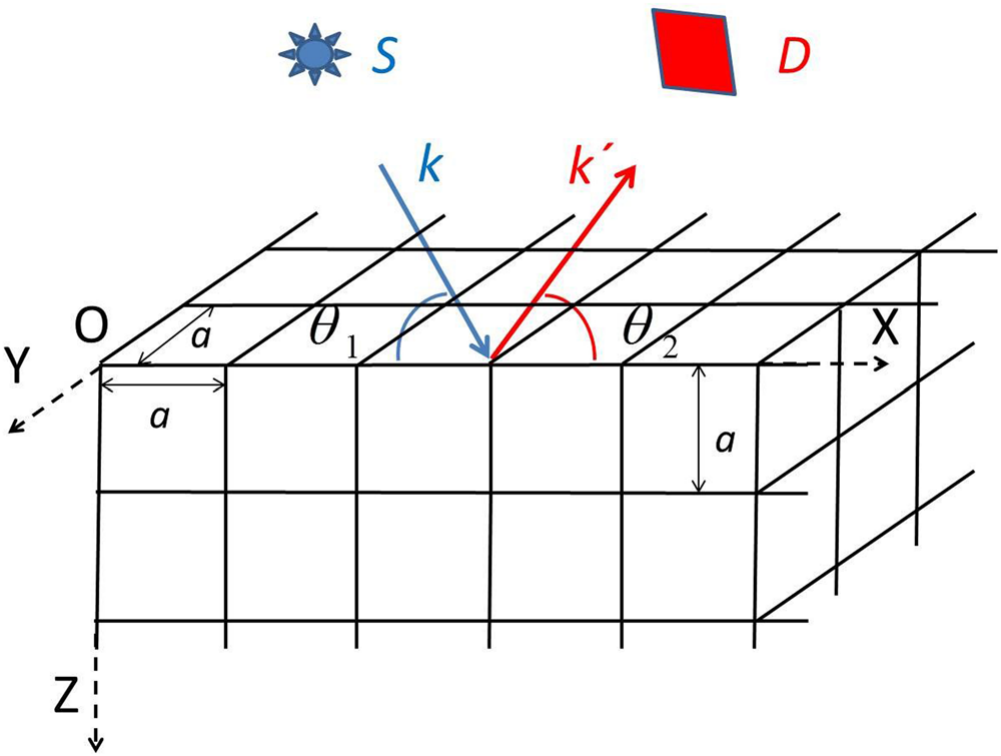
Monochromatic sigma-polarised X-rays from a source S are incident upon a three-dimensional crystalline sample, for which only one of the top corners is shown. Three-dimensional diffraction data is collected by a detector D which samples diffracted intensities over a three-dimensional range of scattering vectors **Q** = **k′** − **k**, about a particular scattering direction. Such diffraction measurements form the data which may then be input into our method for deterministic Bragg CDI, to yield both the structure factor and the displacement field of the crystalline sample.

Let us consider an incident plane monochromatic X-ray wave having sigma polarisation and unit intensity, which illuminates a small imperfect crystal. The angle between the wave vector ***k*** of the incident wave and the X-axis is *θ*_1_ = *θ*_*B*_ + Δ*θ*_1_ (see Fig. 1), where *θ*_*B*_ is the Bragg angle for a symmetrical (00 L) reflection, Δ*θ*_1_ is an angular deviation and the Cartesian axes (X,Y,Z) are as defined in the Figure. The scattered wave is registered in the direction of the wave vector ***k***′. The angle between ***k***′ and the X-axis is *θ*_2_ = *θ*_*B*_ + Δ*θ*_2_ (see Fig. 1), where Δ*θ*_2_ is the angular deviation. The wave vector ***k*** and the average wave vector ***k***′ lie in the diffraction plane XOZ and $|{\boldsymbol{k}}|=|{\boldsymbol{k}}^{\prime} |=k=2\pi /\lambda$, where *λ* is the wavelength in vacuum. The plane of detector D is perpendicular to the average vector ***k***′.

For simplicity, the crystalline structure is assumed to have cubic symmetry with lattice constant *a* and parallelepiped shape with dimensions *L*_*x*_, *L*_*y*_ and *L*_*z*_. We consider two-beam diffraction in the coplanar geometry, where the XOY plane is the top surface of the crystal (see Fig. 1).

Because of the small size of this crystal, we employ a kinematical approximation to describe diffraction from this structure. We will start from a well-known expression for the amplitude of the scattered wave (in the Fraunhofer approximation) (see e.g., equation (5.3) in ref. 30):

1 $${E}_{kin}=4\pi {r}_{0}C{E}_{i}\iiint d{\bf{r}}{\boldsymbol{^{\prime} }}[-\frac{1}{4\pi }\,\frac{\exp (ik|{\bf{r}}-{\bf{r}}{\boldsymbol{^{\prime} }}|)}{|{\bf{r}}-{\bf{r}}{\boldsymbol{^{\prime} }}|}]\rho ({\bf{r}}{\boldsymbol{^{\prime} }})\exp (i{\bf{k}}\cdot {\bf{r}}{\boldsymbol{^{\prime} }}),$$

where *r*_0_ is the classical electron radius, the incident amplitude *E*_*i*_ = 1 for the illuminating unit intensity wave, *ρ*(**r′**) is the electron density, *C* = sin*ϕ* is the linear polarisation factor, and *ϕ* is the angle between the polarisation vector of the incident wave and the position vector **r** of the observation point. As we consider sigma polarised waves, *C* = 1. If one uses standard approximations for far-field diffraction, namely $k|{\bf{r}}-{\bf{r}}{\boldsymbol{^{\prime} }}|\approx kr-{\bf{k}}{\boldsymbol{^{\prime} }}\cdot {\bf{r}}{\boldsymbol{^{\prime} }}$ for the exponent and $|{\bf{r}}-{\bf{r}}{\boldsymbol{^{\prime} }}|\approx r$ for the denominator in (1), then equation (1) can be rewritten as:

2 $${E}_{kin}=-{r}_{0}\,\frac{\exp (ikr)}{r}\iiint d{\bf{r}}{\boldsymbol{^{\prime} }}\rho ({\bf{r}}{\boldsymbol{^{\prime} }})\exp (-i({\bf{k}}{\boldsymbol{^{\prime} }}-{\bf{k}})\cdot {\bf{r}}{\boldsymbol{^{\prime} }})={A}_{e}\iiint d{\bf{r}}{\boldsymbol{^{\prime} }}\rho ({\bf{r}}{\boldsymbol{^{\prime} }})\exp (-i{\bf{Q}}\cdot {\bf{r}}{\boldsymbol{^{\prime} }}),$$

where **Q** = **k′** − **k** is the scattering vector and ${A}_{e}=-{r}_{0}\frac{\exp (ikr)}{r}$ is a spherical-wave envelope.

The electron density in perfect crystals is a three-dimensional (3D) periodic function, therefore we can further modify (2) (cf. equation (2) in ref. 40):

3 $${E}_{kin}={A}_{e}\sum _{p=1}^{N}{F}_{p}({\bf{Q}})\exp (-i{\bf{Q}}\cdot ({{\bf{R}}}_{p}+{{\bf{u}}}_{p})),$$

where **R**_*p*_ defines the position of the *p*-th cell in an ideal 3D periodic lattice, **u**_*p*_ describes a shift of the *p*-th cell from its position **R**_*p*_, defined in the ideal 3D periodic lattice, ${F}_{p}({\bf{Q}})=\sum _{j=1}^{S}\,{f}_{pj}({\bf{Q}})\exp (-i{\bf{Q}}\cdot {{\bf{r}}}_{pj})$ is the structure factor of the *p*-th cell, **r**_*pj*_ is the position of the *j*-th atom in the *p*-th cell, and ${f}_{pj}({\bf{Q}})=\iiint {\rho }_{pj}({\bf{r}}{\boldsymbol{^{\prime} }})\exp (-i{\bf{Q}}\cdot {\bf{r}}{\boldsymbol{^{\prime} }})d{\bf{r}}{\boldsymbol{^{\prime} }}$ is the atomic scattering factor of the *j*-th atom in the *p*-th cell. It should be noted that we can choose the cell in (3) as an elementary cell, or we can choose this cell as a combination of elementary cells. In the latter case, which is used in our simulations, ${F}_{p}({\bf{Q}})=\sum _{l=1}^{R}{F}_{el}({\bf{Q}})\exp (-i{\bf{Q}}\cdot {{\bf{r}}}_{l})={F}_{el}({\bf{Q}})Z({\bf{q}})$, where *F*_*el*_ is the structure factor of an elementary cell, **r**_*l*_ defines the position of these elementary cells within the *p*-th cell and $Z({\bf{q}})=\sum _{l=1}^{R}\exp (-i{\bf{Q}}\cdot {{\bf{r}}}_{l})=\sum _{l=1}^{R}\exp (-i{\bf{q}}\cdot {{\bf{r}}}_{l})$ is an interference function. Here we (i) assume that all elementary cells in the *p*-th cell are identical, (ii) neglect the effect of shifting the *l*-th elementary cell from its position (in the *p*-th cell), defined in the ideal 3D periodic lattice, and (iii) represent the scattering vector **Q** as **Q** = **h** + **q**, where (iv) **h** is the reciprocal lattice vector for a chosen reflection (e.g., (00 L) in our case) and (v) **q** is the deviation of the scattering vector **Q** from the reciprocal lattice vector **h**. Using the representation of the *p*-th cell as a combination of elementary cells significantly simplifies the simulation procedure because this diminishes the computer memory requirements for the computer simulation and speeds up calculations. This approach was also intrinsically used in iterative BCDI approaches (for a recent review, see e.g. ref. 41 and references therein).

We can further simplify equation (3):

4 $$\begin{array}{rcl}{E}_{kin} & = & {A}_{e}\sum _{p=1}^{N}{F}_{p}({\bf{Q}})\exp (-i{\bf{q}}\cdot {{\bf{R}}}_{p})\exp (-i{\bf{Q}}\cdot {{\bf{u}}}_{p})\\  & \approx  & {A}_{e}\sum _{p=1}^{N}{F}_{p}({\bf{Q}})\exp (-i{\bf{q}}\cdot {{\bf{R}}}_{p})\exp (-i{\bf{h}}\cdot {{\bf{u}}}_{p}).\end{array}$$

Here we have used the fact that **h** · **R**_*p*_ = 2*πl*, where *l* is an integer and **Q** · **u**_*p*_ ≈ **h** · **u**_*p*_ in the vicinity of the reciprocal point **h**.

To proceed further, we divide the expression for the scattered wave given in (4) into two parts. The first part, ${E}_{kin}^{id}$, describes scattering from an ideal (i.e., without deformations and defects), although space-limited, 3D crystal of parallelepiped shape:

5 $${E}_{kin}^{id}={A}_{e}\sum _{p=1}^{N}{F}_{p}^{id}({\bf{Q}})\exp (-i{\bf{q}}\cdot {{\bf{R}}}_{p}),$$

where ${F}_{p}^{id}$ is the structure factor of the *p*-th cell in the case of an ideal crystal. The structure factor ${F}_{p}^{id}({\bf{Q}})$ can be connected to the appropriate Fourier component of polarisability, ${\chi }_{h}^{id}$, of an ideal crystalline structure (comp. eq. 2.37 in ref. 42) corresponding to the reciprocal lattice vector **h**:

6 $${F}_{p}^{id}({\bf{Q}})=-\frac{\pi {V}_{cell}}{{r}_{0}{\lambda }^{2}}\,{\chi }_{h}^{id}Z({\bf{q}}),$$

where *V*_*cell*_ is the volume of the unit cell. Then (5) can be rewritten in the following form:

7 $${E}_{kin}^{id}=-{A}_{e}\frac{\pi {V}_{cell}}{{r}_{0}{\lambda }^{2}}\,{\chi }_{h}^{id}Z({\bf{q}})\sum _{p=1}^{N}\exp (-i{\bf{q}}\cdot {{\bf{R}}}_{p})={B}_{e}{\chi }_{h}^{id}Z({\bf{q}})\sum _{p=1}^{N}\exp (-i{\bf{q}}\cdot {{\bf{R}}}_{p}),$$

where $${B}_{e}=-{A}_{e}\frac{\pi {V}_{cell}}{{r}_{0}{\lambda }^{2}}=\frac{\pi {V}_{cell}}{{\lambda }^{2}}\,\frac{\exp (ikr)}{r}$$.

The second part of equation (4), ${E}_{kin}^{def}$, is the difference between ${E}_{kin}$ and ${E}_{kin}^{id}$:

8 $$\begin{array}{rcl}{E}_{kin}^{def} & = & {B}_{e}Z({\bf{q}})[\sum _{p=1}^{N}{\chi }_{h}^{p}\exp (-i{\bf{q}}\cdot {{\bf{R}}}_{p})\exp (-i{\bf{h}}\cdot {{\bf{u}}}_{p})-{\chi }_{h}^{id}\sum _{p=1}^{N}\exp (-i{\bf{q}}\cdot {{\bf{R}}}_{p})]\\  & = & {B}_{e}Z({\bf{q}})\sum _{p=1}^{N}\exp (-i{\bf{q}}\cdot {{\bf{R}}}_{p})[{\chi }_{h}^{p}\exp (-i{\bf{h}}\cdot {{\bf{u}}}_{p})-{\chi }_{h}^{id}]\\  & = & {B}_{e}{\chi }_{h}^{id}Z({\bf{q}})\sum _{p=1}^{N}\exp (-i{\bf{q}}\cdot {{\bf{R}}}_{p})[{\beta }_{h}^{p}\exp (-i{\bf{h}}\cdot {{\bf{u}}}_{p})-1]\end{array},$$

where ${\beta }_{h}^{p}={\chi }_{h}^{p}/{\chi }_{h}^{id}$ and ${\chi }_{h}^{p}$ is the polarisability for the *p*-th cell, which can be, for instance, zero, if there is no cell in the *p*-th position. Note that we do not consider forbidden reflections in this paper, therefore the function ${\beta }_{h}^{p}$ is always well-defined.

Now we combine our two parts of the scattering amplitude:

9 $$\begin{array}{rcl}{E}_{kin} & = & {E}_{kin}^{id}+{E}_{kin}^{def}\\  & = & {B}_{e}{\chi }_{h}^{id}Z({\bf{q}})\sum _{p=1}^{N}\exp (-i{\bf{q}}\cdot {{\bf{R}}}_{p})+{B}_{e}{\chi }_{h}^{id}Z({\bf{q}})\\  &  & \times \,\sum _{p=1}^{N}\exp (-i{\bf{q}}\cdot {{\bf{R}}}_{p})[{\beta }_{h}^{p}\exp (-i{\bf{h}}\cdot {{\bf{u}}}_{p})-1]\\  & = & {B}_{e}{\chi }_{h}^{id}\frac{\sin ({q}_{x}a{N}_{x}/2)}{\sin ({q}_{x}a/2)}\,\frac{\sin ({q}_{y}a{N}_{y}/2)}{\sin ({q}_{y}a/2)}\,\frac{\sin ({q}_{z}a{N}_{z}/2)}{\sin ({q}_{z}a/2)}\\  &  & \times \,{e}^{-i{q}_{x}a({N}_{x}-1)/2}\,{e}^{-i{q}_{y}a({N}_{y}-1)/2}\,{e}^{-i{q}_{z}a({N}_{z}-1)/2}\\  &  & +\,{B}_{e}{\chi }_{h}^{id}Z({\bf{q}})\sum _{p=1}^{N}\exp (-i{\bf{q}}\cdot {{\bf{R}}}_{p})[{\beta }_{h}^{p}\exp (-i{\bf{h}}\cdot {{\bf{u}}}_{p})-1]\\  & \approx  & {B}_{e}{\chi }_{h}^{id}{N}_{x}{N}_{y}{N}_{z}\frac{\sin ({q}_{x}a{N}_{x}/2)}{({q}_{x}a{N}_{x}/2)}\,\frac{\sin ({q}_{y}a{N}_{y}/2)}{({q}_{y}a{N}_{y}/2)}\,\frac{\sin ({q}_{z}a{N}_{z}/2)}{({q}_{z}a{N}_{z}/2)}\\  &  & {e}^{-i{q}_{x}a({N}_{x}-1)/2}\,{e}^{-i{q}_{y}a({N}_{y}-1)/2}\,{e}^{-i{q}_{z}a({N}_{z}-1)/2}\\  &  & +\,{B}_{e}{\chi }_{h}^{id}Z({\bf{q}})\sum _{p=1}^{N}\exp (-i{\bf{q}}\cdot {{\bf{R}}}_{p})[{\beta }_{h}^{p}\exp (-i{\bf{h}}\cdot {{\bf{u}}}_{p})-1].\end{array}$$

All components of the first term in (9) are well known, therefore this part of the total scattering amplitude can be used in our deterministic approach to reconstruct the second term containing two unknown functions, namely ${\beta }_{h}^{p}$ and **u**_*p*_. The term ${\beta }_{h}^{p}$ is, in general, a complex function. As we have two unknown functions we need, in general, at least two independent sets of data. For instance, these two sets can be collected using different orders of reflection: with the vectors **h** and **h**t, where t is an integer. This allows one to distinguish between the phase component relating to ${\beta }_{h}^{p}$ and to the displacement field **u**_*p*_. However, if the imaginary component of ${\beta }_{h}^{p}$ is small in comparison to its real component (e.g., in the case of centre-symmetrical reflections), one can neglect the phase component of ${\beta }_{h}^{p}$ in comparison to the phase shift caused by the displacement field **u**_*p*_. Then one set of data is enough to recover **u**_*p*_ and the real component of ${\beta }_{h}^{p}$. Also in equation (9) we can take into account that in BCDI experiments (see e.g., ref. 43), angular deviations from the Bragg position are small, therefore $\sin ({q}_{x,y,z}a/2)\approx {q}_{x,y,z}a/2$.

The far-field intensity *I*_*kin*_ (without noise) is:

10 $$\begin{array}{ccc}{I}_{kin}/({|{B}_{e}|}^{2}{|{\chi }_{h}^{id}|}^{2}) & = & {\{{N}_{x}{N}_{y}{N}_{z}\frac{\sin ({q}_{x}a{N}_{x}/2)}{{q}_{x}a{N}_{x}/2}\frac{{\rm{s}}{\rm{i}}{\rm{n}}({q}_{y}a{N}_{y}/2)}{{q}_{y}a{N}_{y}/2}\frac{{\rm{s}}{\rm{i}}{\rm{n}}({q}_{z}a{N}_{z}/2)}{{q}_{z}a{N}_{z}/2}\}}^{2}\\  &  & +\,{N}_{x}{N}_{y}{N}_{z}\frac{\sin ({q}_{x}a{N}_{x}/2)}{{q}_{x}a{N}_{x}/2}\frac{\sin ({q}_{y}a{N}_{y}/2)}{{q}_{y}a{N}_{y}/2}\frac{\sin ({q}_{z}a{N}_{z}/2)}{{q}_{z}a{N}_{z}/2}\\  &  & \times \,{e}^{i{q}_{x}a({N}_{x}-1)/2}{e}^{i{q}_{y}a({N}_{y}-1)/2}{e}^{i{q}_{z}a({N}_{z}-1)/2}\\  &  & \times \,Z({\bf{q}})\sum _{p=1}^{N}\exp (-i{\bf{q}}\cdot {{\bf{R}}}_{p})[{\beta }_{h}^{p}\exp (-i{\bf{h}}\cdot {{\bf{u}}}_{p})-1]\\  &  & +\,{N}_{x}{N}_{y}{N}_{z}\frac{\sin ({q}_{x}a{N}_{x}/2)}{{q}_{x}a{N}_{x}/2}\frac{\sin ({q}_{y}a{N}_{y}/2)}{{q}_{y}a{N}_{y}/2}\frac{\sin ({q}_{z}a{N}_{z}/2)}{{q}_{z}a{N}_{z}/2}\\  &  & \times \,{e}^{-i{q}_{x}a({N}_{x}-1)/2}{e}^{-i{q}_{y}a({N}_{y}-1)/2}{e}^{-i{q}_{z}a({N}_{z}-1)/2}\\  &  & \times \,{Z}^{\ast }({\bf{q}})\sum _{p=1}^{N}\exp (i{\bf{q}}\cdot {{\bf{R}}}_{p})[{({\beta }_{h}^{p})}^{\ast }\exp (i{\bf{h}}\cdot {{\bf{u}}}_{p})-1]\\  &  & +\,{|Z({\bf{q}})\sum _{p=1}^{N}\exp (-i{\bf{q}}\cdot {{\bf{R}}}_{p})[{\beta }_{h}^{p}\exp (-i{\bf{h}}\cdot {{\bf{u}}}_{p})-1]|}^{2}.\end{array}$$

To proceed further we introduce the auxiliary function *U*(*x*, *y*, *z*), which is similar to that introduced in ref. 36 and which is able to provide a closed-form solution to the BCDI inverse problem of reconstructing ${\beta }_{h}^{p}\exp (-i{\bf{h}}\cdot {{\bf{u}}}_{p})$:

11 $$U({\bf{R}}=(x,y,z))={|{B}_{e}|}^{-2}{|{\chi }_{h}^{id}|}^{-2}\frac{1}{{(2\pi )}^{3/2}}\iiint {q}_{x}{q}_{y}{q}_{z}{\hat{I}}_{kin}\exp (i{q}_{x}x+i{q}_{y}y+i{q}_{z}z)d{q}_{x}d{q}_{y}d{q}_{z}.$$

**Figure 2:**
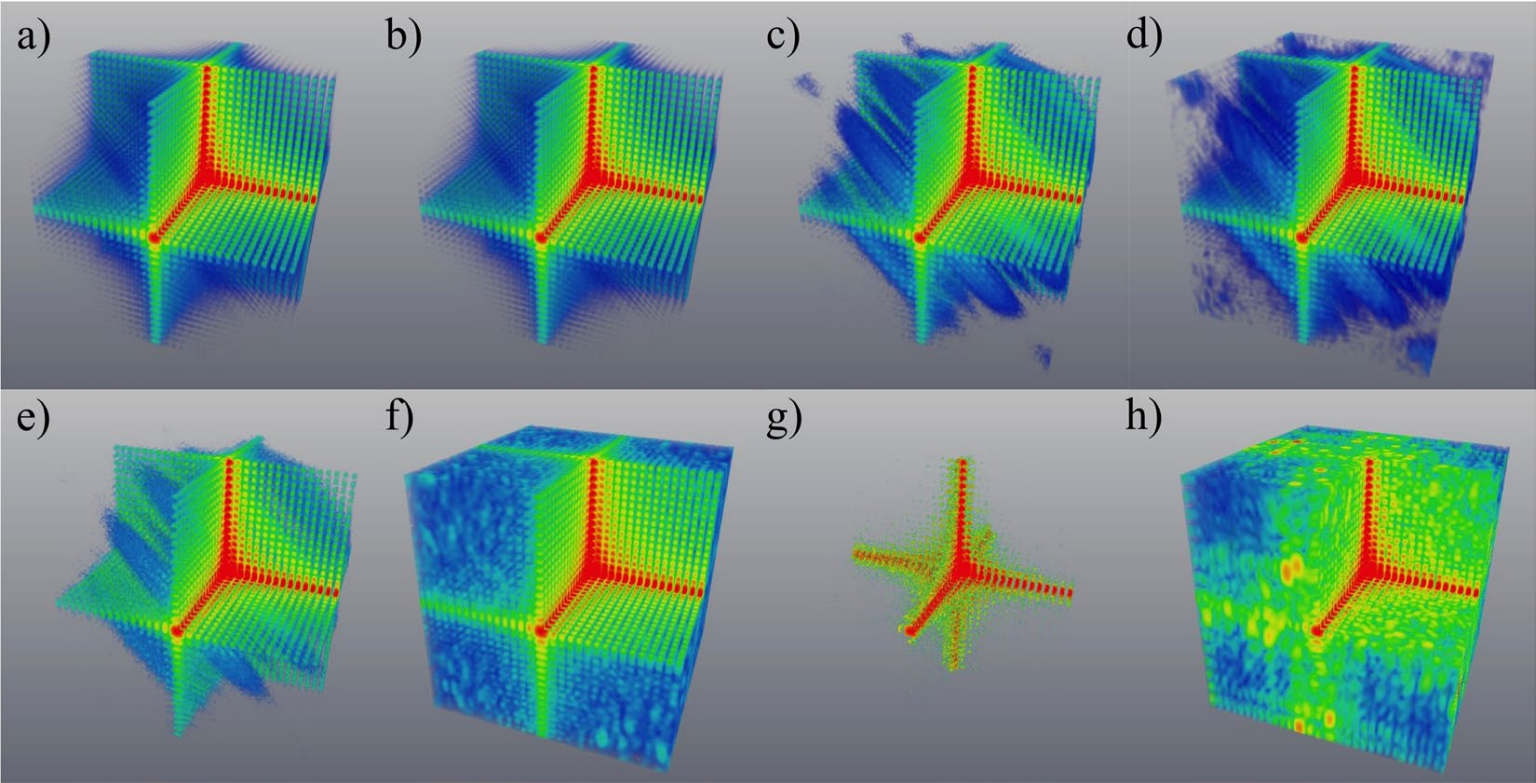
Simulated 3D distributions of diffracted intensity for the case of weak parabolic displacement field and weak spherical inclusions, with no imperfections in the lower (“reference”) half of the crystal. All intensities are shown on a logarithmic scale, with the front octant subtracted to visualise the interior. The coefficient *γ*, which is inversely proportional to the radius of curvature introduced into the simulated crystal structure, is chosen to yield a maximum phase shift of 0.25π. The quantity ${\beta }_{h}^{p}$, namely the ratio of polarisability (corresponding to the reciprocal vector **h**) of the p-th cell to polarisability of the cell in the “reference” part of the crystal, is equal to 0.9 for the spherical inclusions modelled here. (**a**,**c**,**e**) and (**g**) - the intensity simulated using the original model for the phase and amplitude; (**b**,**d**,**f**) and (**h**) the intensity simulated using the reconstructed values for the phase and amplitude; (**a**,**b**) – no noise; (**c**,**d**) – maximum intensity of 10^9^ photons per voxel; (**e**,**f**) – maximum intensity of 10^8^ photons per voxel; (**g**,**h**) – maximum intensity of 10^5^ photons per voxel. In all panels, Fourier-space coordinates q_x_, q_y_ and q_z_ lie within the range ±1.96 × 10^−2^ nm^−1^. See Supplementary Movie 2 for an animation corresponding to this Figure.

Here ${\hat{I}}_{kin}$ (shown in Fig. 2 for a “weak” object) is the intensity *I*_*kin*_ with added Poisson noise. For real experimental data ${\hat{I}}_{kin}$ is proportional to the registered intensity. Thus the auxiliary function *U*(*x*, *y*, *z*) is simply formed by multiplying the measured noisy far-field diffracted intensity ${\hat{I}}_{kin}({q}_{x},{q}_{y},{q}_{z})$ by the product of Fourier coordinates *q*_*x*_ · *q*_*y*_ · *q*_*z*_, and then taking the 3D inverse Fourier transform of the result.

As shown in detail in the supplementary section, the auxiliary function *U*(*x*, *y*, *z*) reduces to

12 $$U({\bf{R}}=(x,y,z))=A+\sum _{j=1}^{8}({B}_{j}+{C}_{j})+D.$$

**Figure 3:**
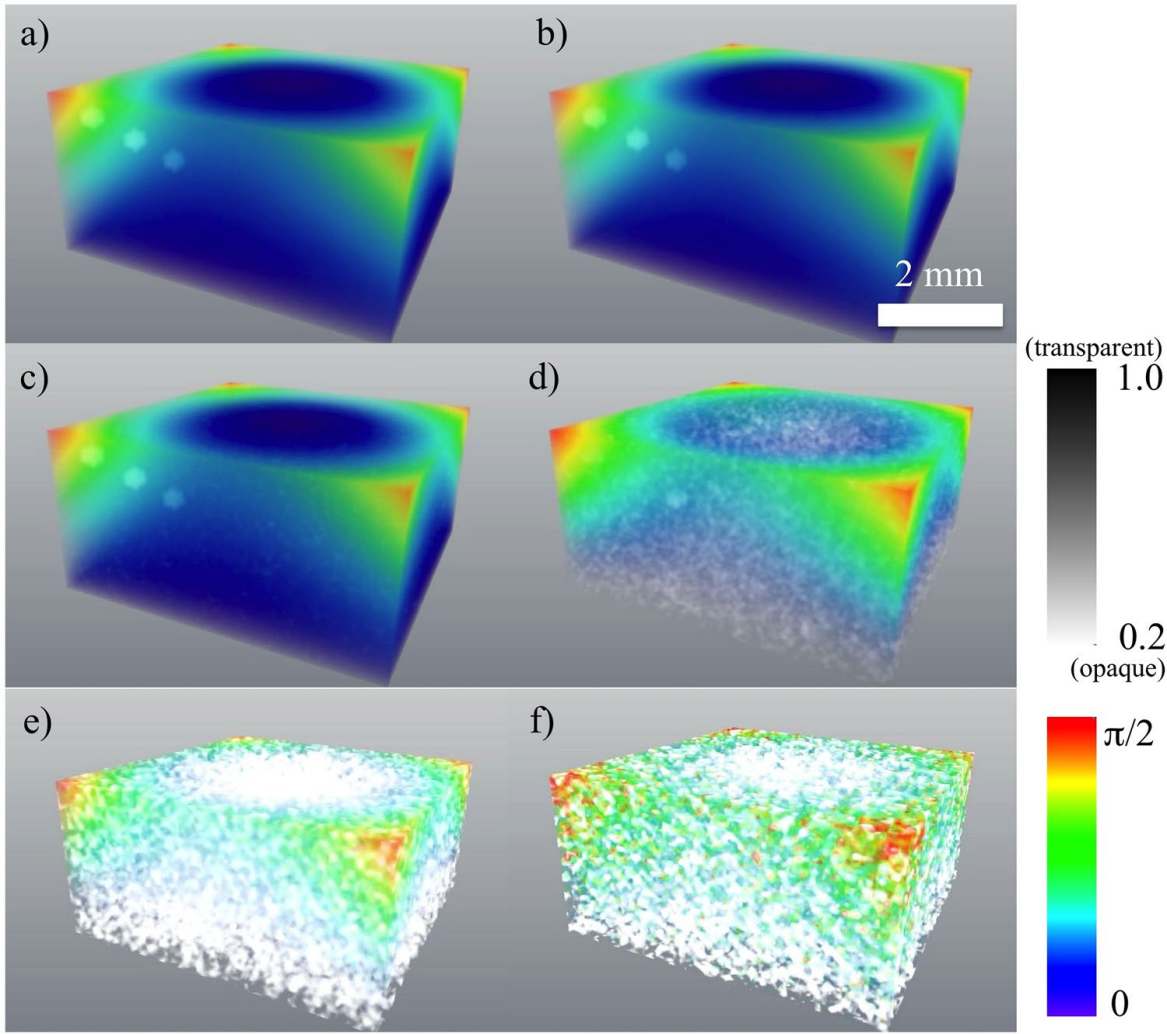
Reconstruction of the crystal in the case of weak parabolic displacement field and weak spherical inclusions, for varying levels of noise, and no imperfections in the “reference” half of the crystal. The amplitude (shown in grey scale) and phase (shown in colour scale). The coefficient *γ* is chosen to yield a maximum phase shift of 0.25π. ${\beta }_{h}^{p}=0.9$ for spherical inclusions. (**a**) – the original model, (**b**) – the reconstruction in absence of noise, (**c**) – the reconstruction with maximum intensity of 10^9^ photons per voxel, (**d**) – the reconstruction with maximum intensity of 10^8^ photons per voxel, (**e**) – the reconstruction with maximum intensity of 10^6^ photons per voxel, (**f**) – the reconstruction with maximum intensity of 10^5^ photons per voxel. See Supplementary Figure 3 for a 2D slice going through the innermost sphere and Supplementary Movie 3 for an animation corresponding to this Figure.

**Figure 4:**
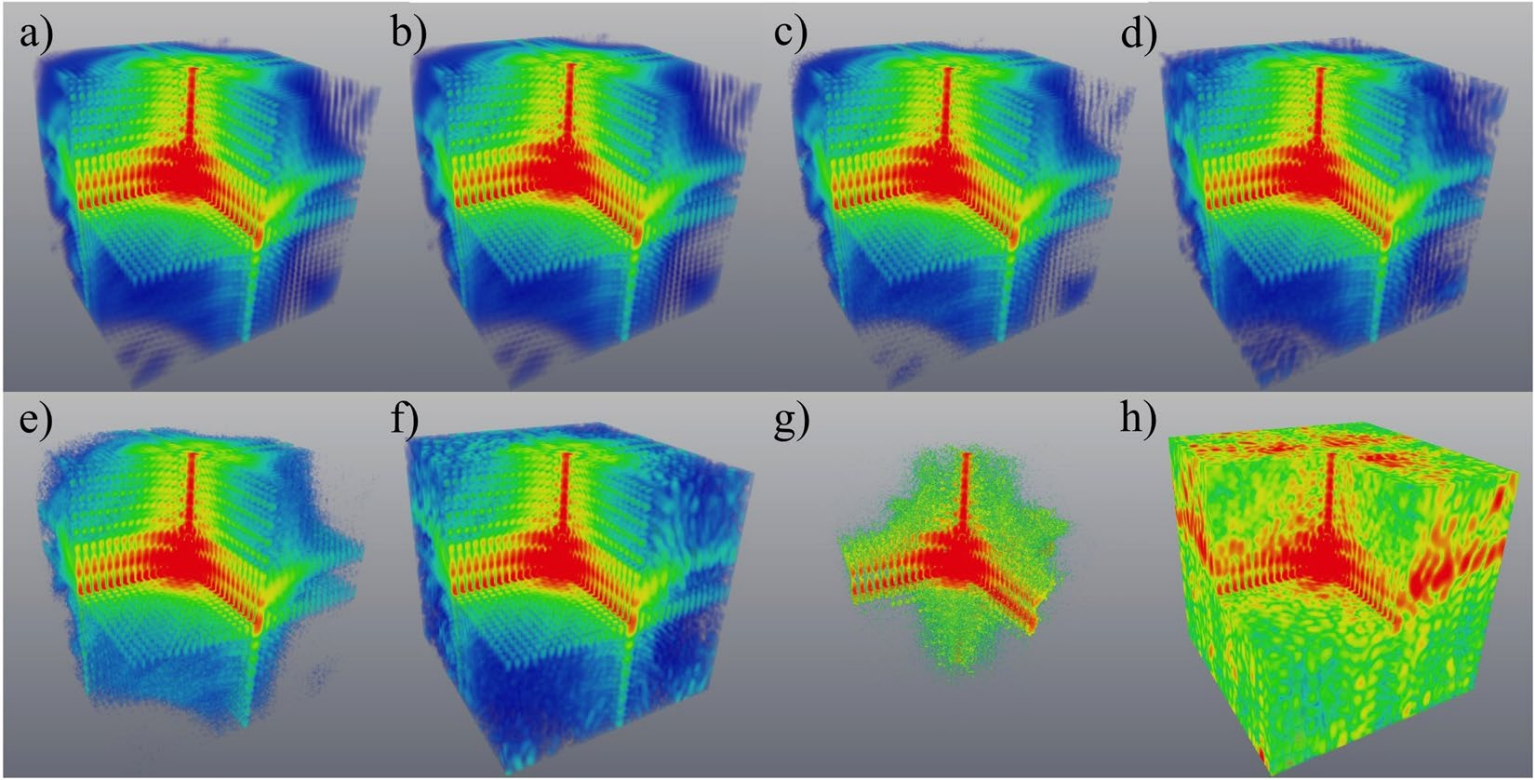
Simulated 3D distributions of diffracted intensity for the case of strong parabolic displacement field and strong spherical inclusions, with no imperfections in the lower (“reference”) half of the crystal. All intensities are shown on a logarithmic scale, with the front octant subtracted to visualise the interior. The coefficient *γ* is chosen to yield a maximum phase shift of 3π. ${\beta }_{h}^{p}=0$ for spherical inclusions. (**a**,**c**,**e**) and (**g**) - the intensity simulated using the original model for the phase and amplitude; (**b**,**d**,**f**) and (**h**) the intensity simulated using the reconstructed values for the phase and amplitude; (**a**,**b**) – no noise; (**c**) and (**d**) – maximum intensity of 10^9^; (**e**,**f**) – maximum intensity of 10^8^; (**g**,**h**) – maximum intensity of 10^5^. In all panels, Fourier-space coordinates q_x_, q_y_ and q_z_ lie within the range ±1.96 × 10^−2^ nm^−1^. See Supplementary Movie 4 for an animation corresponding to this Figure.

**Figure 5:**
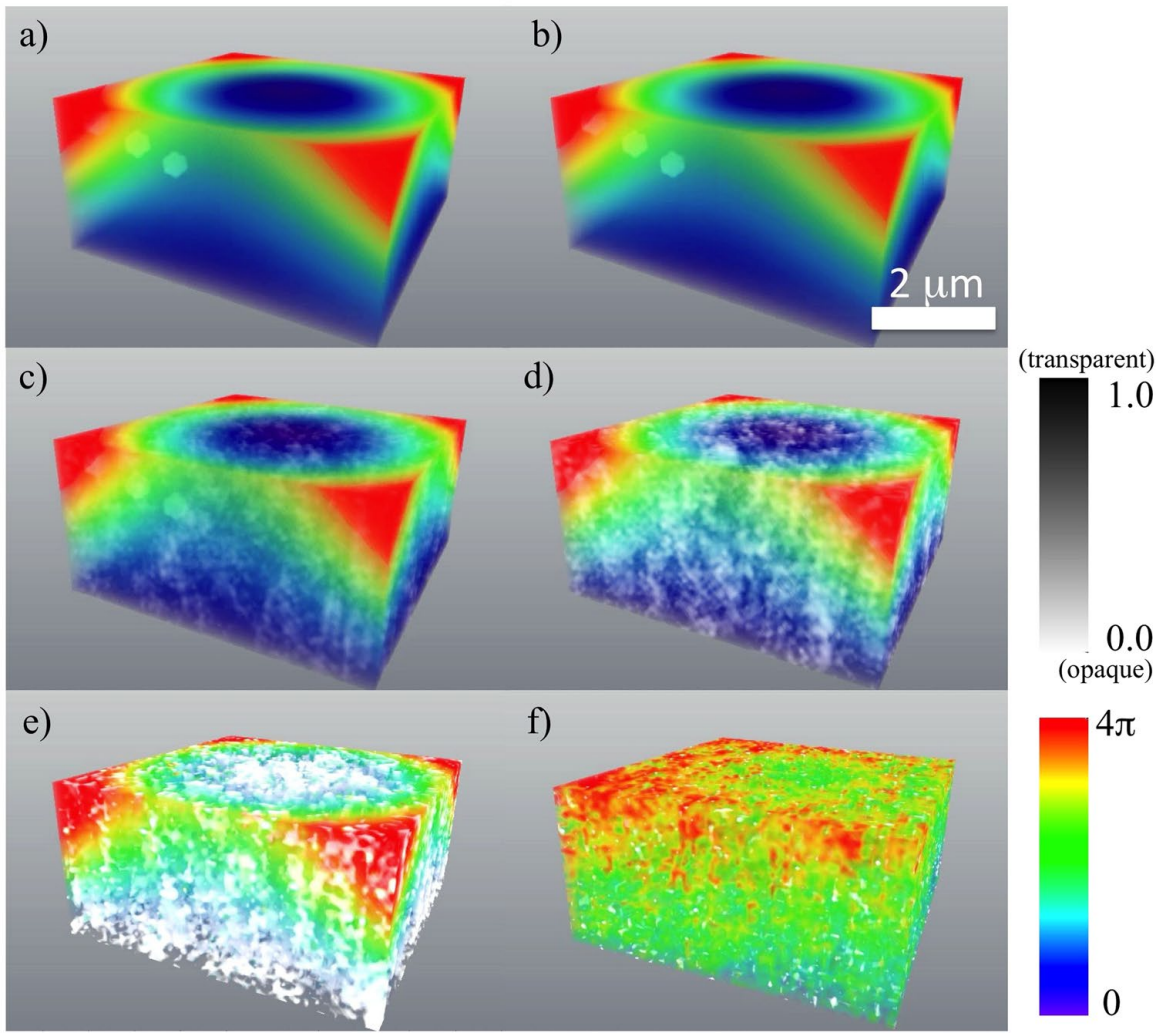
Reconstruction of the crystal in the case of strong parabolic displacement field and strong spherical inclusions, for varying levels of noise, and no imperfections in the “reference” half of the crystal. The amplitude (shown in grey scale) and phase (shown in colour scale). The coefficient *γ* is chosen to yield a maximum phase shift of 3π. ${\beta }_{h}^{p}=0$ for spherical inclusions. (**a**) – the original model, (**b**) – the reconstruction in absence of noise, (**c**) – the reconstruction with maximum intensity of 10^9^, (**d**) – the reconstruction with maximum intensity of 10^8^, (**e**) – the reconstruction with maximum intensity of 10^6^, (**f**) – the reconstruction with maximum intensity of 10^5^. See Supplementary Figure 5 for a 2D slice going through the most inner sphere and Supplementary Movie 5 for an animation corresponding to this Figure.

Here, *A* is a term proportional to the shape function of the sample, the *B*_*j*_ and *C*_*j*_ terms are 16 spatially-translated independent reconstructions of the unknown field ${\beta }_{h}^{p}\exp (-i{\bf{h}}\cdot {{\bf{u}}}_{p})$ (the phase and amplitude of which are shown in Fig. 3 for a “weak” object) or its complex conjugate, and *D* is related to the derivative of the cross-correlation of the object. Further details are given in both the supplementary section, and the main text below. Note that the intensity ${\hat{I}}_{kin}$ and the phase and amplitude for a “strong” phase object are shown in Figs 4 and 5, respectively.

The previously-mentioned spatially translated reconstructions are spatially separated from the derivative of the cross-correlation function, namely the term *D* in (12), placed in the centre. The term *A* equals constants within each of 8 octants of the reconstruction. The condition of the separation, namely having the reconstructions *B*_*j*_ and *C*_*j*_ each exhibiting no overlap with any other terms in (12), is that the part of the crystal with deformations and defects should be smaller than 1/2 of the total volume. This key restriction on our method is similar to one obtained in a deterministic variant for transmission-based 2D coherent diffractive imaging^36, 44, 45^. In this paper, for simplicity, we can neglect the imaginary component (as is usually done in BCDI, see e.g., ref. 41) of the function ${\beta }_{h}^{p}={\chi }_{h}^{p}/{\chi }_{h}^{id}$ because the imaginary parts of the functions ${\chi }_{h}^{p}$ and ${\chi }_{h}^{id}$ are typically significantly smaller than the corresponding real parts. Then the reconstructed function $\psi ({\bf{R}})\approx \mathrm{Re}({\beta }_{h}^{p})\exp (-i{\bf{h}}\cdot {{\bf{u}}}_{p})$ directly supplies information about ${\rm{Re}}({\beta }_{h}^{p})=|\psi ({\bf{R}})|$ and $\exp (-i{\bf{h}}\cdot {{\bf{u}}}_{p})=\psi ({\bf{R}})/|\psi ({\bf{R}})|$. However, if **h** · **u**_*p*_ > 2*π* this causes problems^38^ in determining the displacement field **u** because of the well-known problems of phase unwrapping.

To solve the problem of the phase unwrapping, we introduce three modified auxiliary functions *W*_*x*,*y*,*z*_(*x*, *y*, *z*), which are essentially the appropriate partial derivatives of the function *U*(*x*, *y*, *z*) with respect to the x (or y, or z) coordinate:

13 $${W}_{x,y,z}({\bf{R}}=(x,y,z))={\partial }_{x,y,z}U(x,y,z)={\partial }_{x,y,z}[A+\sum _{j=1}^{8}({B}_{j}+{C}_{j})+D].$$

As discussed above, the functions *B*_1,2,3,4,5,6,7,8_ contain the spatially translated variants of the reconstructed function $\psi ({\bf{R}})={\beta }_{h}^{p}\exp (-i{\bf{h}}\cdot {{\bf{u}}}_{p})$. Thus their derivatives, ${\partial }_{x,y,z}\psi ({\bf{R}})$, produced by *W*_*x*,*y*,*z*_(*x*, *y*, *z*), will also be spatially separated. As we already reconstructed the function $\psi ({\bf{R}})={\beta }_{h}^{p}\exp (-i{\bf{h}}\cdot {{\bf{u}}}_{p})$, we can obtain the appropriate partial derivatives of its phase:

14 $${\partial }_{x,y,z}(-i{\bf{h}}\cdot {{\bf{u}}}_{p})\approx \frac{{\psi }^{\ast }({\bf{R}})\cdot {\partial }_{x,y,z}\psi ({\bf{R}})-\psi ({\bf{R}})\cdot {\partial }_{x,y,z}{\psi }^{\ast }({\bf{R}})}{2{({\beta }_{h}({\bf{R}}))}^{2}}.$$

This allows us to reconstruct the phase, −**h** · **u**_*p*_, of the complex function $\psi ({\bf{R}})={\beta }_{h}^{p}\exp (-i{\bf{h}}\cdot {{\bf{u}}}_{p})$ from its derivatives. In particular, we use 2D “slices” of partial derivatives^46^. To avoid division by 0 in equation (14) the following regularisation procedure was employed: if the reconstructed amplitude is less than a particular small constant, the phase and its derivatives are all assumed be 0. The physical meaning of this regularisation is obvious. As a small (comparable to noise) reconstructed amplitude indicates that this particular voxel does not produce a meaningful signal, we cannot obtain the phase of a complex function having amplitude close to 0. However, some additional operations, for instance, averaging over neighbouring voxels, can be used to reconstruct the phase (as a continuous function) in voxels having amplitude undistinguishable from noise.

The unwrapped phase, *ϕ*_*unwr*_, reconstructed from its derivatives (see equation (14)) is usually noisier in comparison to the wrapped phase, *ϕ*_*wr*_, reconstructed directly using (11). Therefore, we apply a simple unwrapping algorithm allowing us to unwrap *ϕ*_*wr*_ to obtain the phase *ϕ*:

15 $$\varphi ={\varphi }_{wr}+2\pi \cdot INT\{({\varphi }_{unwr}-{\varphi }_{wr})/2\pi \},$$

where *INT* stands for an operation of finding the nearest integer. If the argument *E* of *INT* is exactly half-integral, then *INT* rounds down to the nearest integer that is smaller than *E*.

### Numerical Results

In our simulations we use the following model for the crystal: a parallelepiped having dimensions *L*_*x*_, *L*_*y*_ and *L*_*z*_, where the z-direction is vertical and XOY is the horizontal plane (cf. Fig. 1). For simplicity, *L*_*x*_ = *L*_*y*_ = *L*_*z*_. The upper half of this crystalline structure contains a “non-ideal” crystalline structure. The “non-idealness” is assumed to be caused by existence of a deformation field, which is specified below, and three spherical inclusions. These inclusions are either “strong” inclusions filled with air (i.e., minimum ${\beta }_{h}^{p}=0$) or “weak” inclusions filled with a material having a smaller structure factor (minimum ${\beta }_{h}^{p}=0.9$). The remainder (apart from the spherical inclusions) of the crystalline structure has ${\beta }_{h}^{p}=1$. The cropped data array of far-field intensity used in reconstructions results in a voxel resolution in real space of 80 × 80 × 80 nm^3^, which is comparable to the resolution demonstrated in ref. 43. However, it should be noted that a better resolution is reported in more recent literature^47, 48^. Demonstration of the applicability of the proposed reconstruction technique for a smaller voxel size is planned for future research.

In the “non-ideal” part of the crystalline object (see Figs 3(a) and 5(a)) we use a model of deformation, which is an extended (3D) variant of the 1D model of the deformation field reported in ref. 38. This parabolic displacement may be associated^38^ with the attachment of the “non-ideal” part of the crystal (*z* ∈ [0, *L*_*z*_/2]) to an “ideal” (i.e., ${\beta }_{h}^{p}\equiv 1$ and **u**(**r**) ≡ 0) part of the crystal (*z* ∈ ]*L*_*z*_/2*, L*_*z*_]) and can be approximated as $\exp (-i{\bf{h}}\cdot {\bf{u}}({\bf{r}}))=\exp (i\gamma [{(x-{L}_{x}/2)}^{2}+{(y-{L}_{y}/2)}^{2}]\cdot [1-2z/{L}_{z}])$ for *z* ∈ [0, *L*_*z*_/2], where *γ* is a constant that is inversely proportional to the radius of curvature. In our simulations we have chosen the values of the coefficient *γ* to yield a maximum phase shift of either 0.25π (“weak” case) or 3π radians (“strong” case) at the edges of the “non-ideal” part of the crystal. The latter “strong” phase limit, namely with phase shift of more than 2π radians, is extremely likely to occur in practice^38^. We note, in this context, that the support-based phasing method, used in the iterative reconstruction procedure reported in ref. 38, was not successful for such large phase shifts. As shown in Table 1 and in Figs 3 and 5, our technique allows reconstruction of the phase even for such a large phase range.

**Table 1:** Error metrics for the real space and Fourier space data, for the case where the lower (“reference”) half of the crystal is assumed to be perfect.

Maximum intensity level	Maximum phase (rad) due to parabolic displacement field	Minimum amplitude due to spherical inclusions	*Normalised RMS error* (*d_ph*) *for reconstructed phase*	*Normalised absolute error* (*r_ph*) *for reconstructed phase*	*Normalised RMS error* (*d_amp*) *for reconstructed amplitude*	*Normalised absolute error* (*r_amp*) *for reconstructed amplitude*	*Error metric* (*χ* ^2^) *in Fourier space*
Infinite (no noise)	0.25	0	0.07	0.04	3 × 10^−13^	9 × 10^−8^	2 × 10^−29^
	0.25	0.9	2 × 10^−14^	1 × 10^−7^	3 × 10^−12^	9 × 10^−8^	2 × 10^−29^
	3	0	0.07	0.04	4 × 10^−13^	8 × 10^−8^	4 × 10^−29^
	3	0.9	3 × 10^−15^	4 × 10^−8^	4 × 10^−12^	8 × 10^−8^	4 × 10^−29^
10^5^	0.25	0	6	2	7 × 10^1^	1	8 × 10^−2^
	0.25	0.9	2	1	2 × 10^2^	1	3 × 10^−2^
	3	0	2	1	7 × 10^1^	1	7 × 10^−1^
	3	0.9	2	1	5 × 10^2^	1	7 × 10^−1^
10^6^	0.25	0	3	1	2 × 10^1^	1	1 × 10^−2^
	0.25	0.9	1	1	5 × 10^1^	3 × 10^−1^	3 × 10^−3^
	3	0	5 × 10^−1^	5 × 10^−1^	2 × 10^1^	1	6 × 10^−2^
	3	0.9	2	1	2 × 10^2^	1	5 × 10^−2^
10^8^	0.25	0	0.2	0.4	2	0.2	1 × 10^−4^
	0.25	0.9	0.05	0.2	5	0.1	6 × 10^−5^
	3	0	0.07	0.1	2	0.2	5 × 10^−4^
	3	0.9	0.02	0.1	15	0.2	5 × 10^−4^
10^9^	0.25	0	0.1	0.2	0.6	0.1	1 × 10^−5^
	0.25	0.9	0.02	0.1	2	0.06	8 × 10^−6^
	3	0	0.07	0.07	0.6	0.1	5 × 10^−5^
	3	0.9	0.005	0.06	5	0.1	4 × 10^−5^

Successive main rows are labelled according to the total number of photons corresponding to the voxel with maximum detected intensity. The brightest voxel, corresponding to the origin of Fourier space, was excluded from the noise adding procedure as the appropriate intensity is multiplied by zero (see equation (11)) during the reconstruction process. Each main row has four sub-cases, corresponding to the parabolic displacement field and spherical inclusions being weak/strong, weak/weak, strong/strong and strong/weak, respectively. For each sub-case, five different error metrics (specified in equations (16), (17) and (18) of the main text) are given.

**Figure 6:**
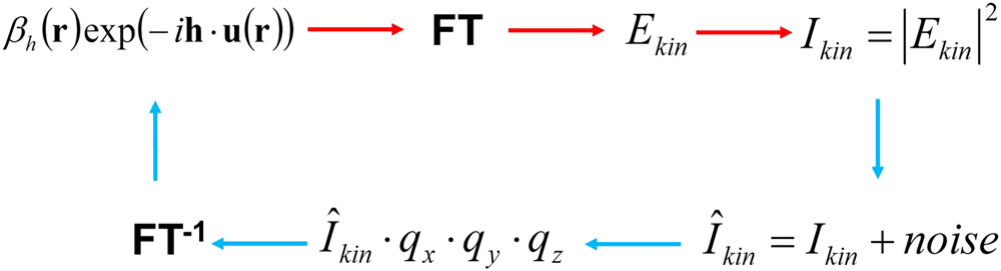
Flow chart of the forward problem of modelling the far-field intensity produced by X-rays illuminating a non-ideal crystal (red arrows) and solution to the associated inverse problem given by our reconstruction procedure as specified by equations (11) and (12) (blue arrows). Here, FT and FT^−1^ stand for the direct and inverse 3D Fourier transforms.

To demonstrate the robustness of the reconstruction algorithm, for which a flow-chart is given in Fig. 6, we incorporate pseudo-random Poisson noise (see e.g., refs 36, 49) in the simulated intensity function. We use four levels of the maximum intensity in our simulations of the intensity with noise, namely 10^5^, 10^6^, 10^8^ and 10^9^ photons per voxel of the diffracted intensity distribution. It should be noted that the brightest voxel, corresponding to the origin *q*_*x*_ = *q*_*y*_ = *q*_*z*_ = 0 of Fourier space, was excluded from the noise adding procedure as the appropriate intensity is multiplied by zero (see equation (11)) during the reconstruction process. Figures 2–5 (with associated Supplementary movies) show the diffracted intensity distributions (on a logarithmic scale) and the original and reconstructed phase and amplitude for two cases: a so-called “weak” object (see Figs 2 and 3) (a maximum phase shift of 0.25π and ${\beta }_{h}^{p}=0.9$ for spherical inclusions) and a “strong” phase object (see Figs 4 and 5) (a maximum phase shift of 3π and ${\beta }_{h}^{p}=0$ for spherical inclusions). The error metrics for other cases (not shown here) can be found in Table 1.

The simulated far-field diffraction patterns for different levels of noise are shown in Figs 2 and 4, where parts (a), (c), (e) and (g) show the intensity simulated using the original model for the phase and amplitude, and parts (b), (d), (f) and (h) show the intensity simulated using the reconstructed values for the phase and amplitude. The former corresponds to the simulated “experimental” intensity, while the latter is typically used in the iterative reconstruction algorithms to estimate the “closeness” of the obtained solution for the phase and amplitude. To estimate errors in the reconstructed functions, with amplitude, ${\beta }_{h}^{p}$, and phase, (−*i* **h** · **u**_*p*_), we use for real space data two metrics^50^, namely a normalised root-mean-square (RMS) error criterion, defined as

16 $$d=\sqrt{\sum {({G}_{ijk}^{rec}-{G}_{ijk}^{ideal})}^{2}/\sum {({G}_{ijk}^{ideal}-\langle {G}^{ideal}\rangle )}^{2}},$$

and a normalised absolute difference,

17 $$r=\sqrt{\sum |{G}_{ijk}^{rec}-{G}_{ijk}^{ideal}|/\sum |{G}_{ijk}^{ideal}|},$$

where ${G}_{ijk}^{ideal}$ and ${G}_{ijk}^{rec}$ are ideal and reconstructed three-dimensional functions, respectively. $\langle {G}^{ideal}\rangle$ is the mean of the original function. Table 1 shows values of criteria *d_ph*, *r_ph* and *d_amp*, *r_amp* for the phase and amplitude, respectively, for different values of maximum phase shift, minimum amplitude in spherical clusters and noise levels. The last column in Table 1 shows the error metric in Fourier space, which is defined as

18 $${\chi }^{2}={{\sum }_{i=1}^{N}(\sqrt{{\hat{I}}_{kin,i}}-\sqrt{I{r}_{i}})}^{2}/{\sum }_{i=1}^{N}({\hat{I}}_{kin,i}),$$

where ${\hat{I}}_{kin,i}$ is the 3D array of the simulated intensity distribution (with added noise) in Fourier space modelled using the original model for the phase and amplitude, and *Ir*_*i*_ is the 3D array of the simulated intensity distribution in Fourier space modelled using the reconstructed (using equations (11–15)) values for the phase and amplitude. This error metric is typically used in iterative reconstruction algorithms^43^. The *χ* ^2^ error metric achieved in iterative reconstruction using real experimental data^43^ is similar to our results (see Table 1) for the maximum intensities of 10^5^ and 10^6^. However, the improved signal-to-noise ratio (SNR) for the maximum intensities of 10^8^ and 10^9^ photons per voxel produced smaller *χ* ^2^ (see Table 1). As expected, the larger SNR of the diffraction data causes better reconstruction of the phase and amplitude (see Table 1).

**Figure 7:**
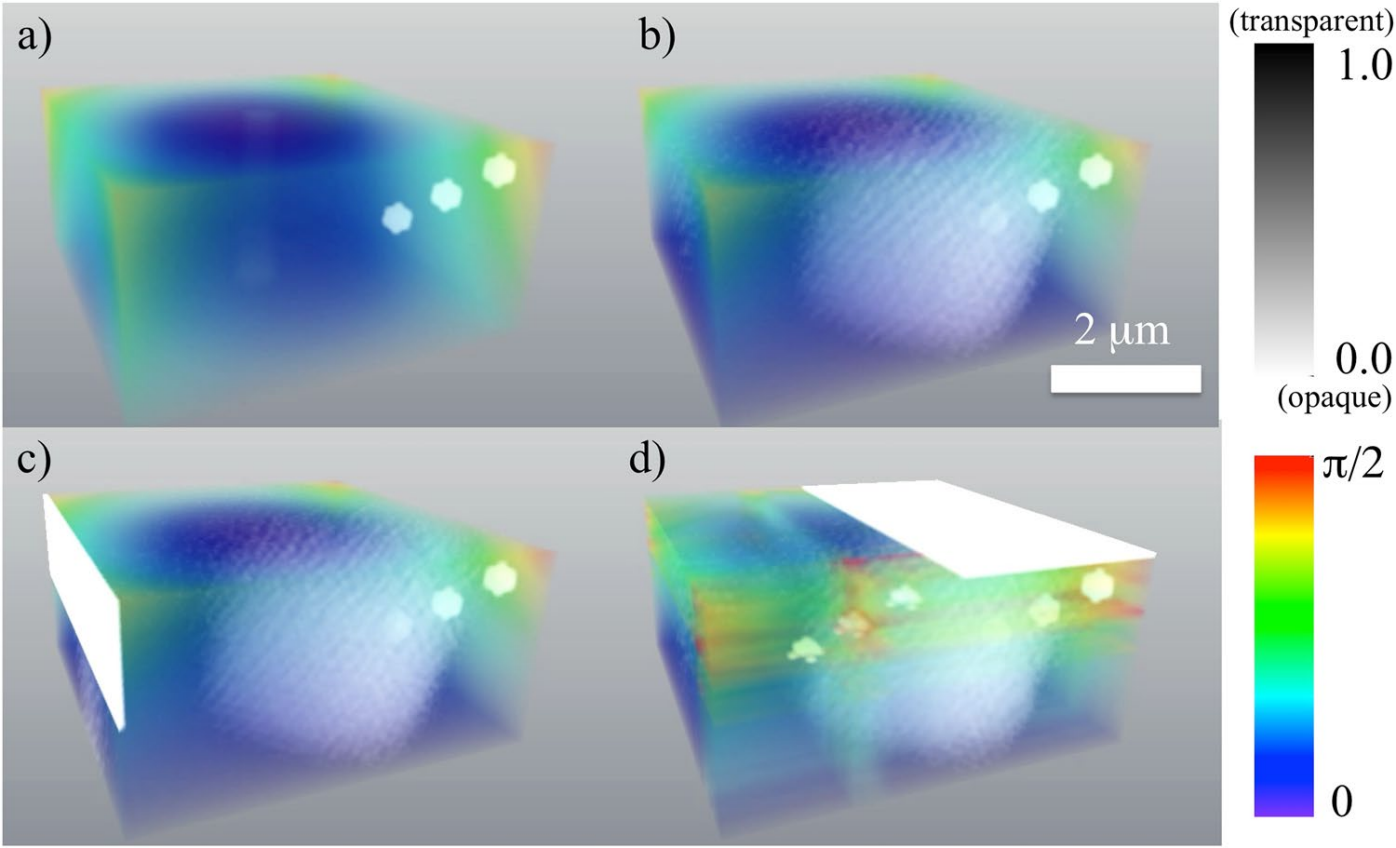
Input versus reconstruction of the crystal in the case of weak parabolic displacement field and strong spherical inclusions, for a fixed level of noise, and three different departures from ideality in the “reference” half of the crystal. The amplitude (shown in grey scale) and phase (shown in colour scale). The coefficient *γ* is chosen to yield a maximum phase shift of 0.25π. ${\beta }_{h}^{p}=0$ for spherical inclusions. The maximum intensity is 10^9^ photons per voxel. (**a**) – the original model for a displacement field throughout the crystal (shown only the upper part of the crystal), (**b**) – the reconstruction for a displacement field throughout the crystal, (**c**) – the reconstruction for a displacement field throughout the crystal when a slice 1 pixel(X) × 16 pixels(Z) × 64 pixels(Y) is removed from the bottom part of the crystal, (**d**) – the reconstruction for a displacement field throughout the crystal when a slice 32 pixels(X) × 1 pixel(Z) × 64 pixels(Y) is removed from the bottom part of the crystal. See Supplementary Figure 7 for a 2D slice going through the most inner sphere and Supplementary Movie 7 for an animation corresponding to this Figure.

We augment the preceding simulations by considering the effectiveness of the method when there is a deviation from ideal conditions, such as when the crystal geometry is not known or is not perfect in the ‘reference’ part of the sample. Accordingly, we test the robustness of our reconstruction technique in the case of the bottom half of the crystal being non-ideal (see Table 2 and Fig. 7). We consider three cases with increasing level of imperfections, all of which may be compared to the ideal case in Fig. 7a. In all cases the maximum intensity is 10^9^ photons per voxel (excluding the origin, for reasons outlined earlier). In the first non-ideal case (see Fig. 7b) the deformation field is extended to the entire crystal according to the previously-stated functional form: $\exp (-i{\bf{h}}\cdot {\bf{u}}({\bf{r}}))=\exp (i\gamma [{(x-{L}_{x}/2)}^{2}+{(y-{L}_{y}/2)}^{2}]\cdot [1-z/{L}_{z}])$ for *z* ∈ [0, *L*_*z*_]. In these simulations (see Fig. 7) we have chosen the values of the coefficient *γ* to yield a maximum phase shift of 0.25π at the edges of the crystal to make displacements in the bottom half of the crystal relatively small. In the second non-ideal case the reconstruction (see Fig. 7c) is done for the same displacement field as in the first case above when a slice having dimensions 1 pixel(X) × 16 pixels(Z) × 64 pixels(Y) is removed from the bottom part of the crystal. In the third (most extreme) non-ideal case (see Fig. 7d) the reconstruction is done for the displacement field (same as in the first case) when a slice 32 pixels(X) × 1 pixels(Z) × 64 pixels(Y) is removed from the bottom part of the crystal. One voxel in real space has dimensions 80 nm × 80 nm × 80 nm. As shown in Table 2 and in Fig. 7, our technique allows reconstruction (with artefacts) of the phase and amplitude even for the case of non-ideal bottom half of the crystal. The stronger the deteriorations in the bottom half of the crystal are, the more artefacts are observed (see Fig. 7) and the worse the error metrics become (see Table 2). The error metrics shown in Table 2 are similar to the case of ideal bottom half of the crystal if the maximum intensity is of 10^8^ photons per voxel (see Table 1). However, there are visible artefacts in the reconstructed samples (see Fig. 7). See also Supplementary Movie 7 for an animation corresponding to Fig. 7.

**Table 2:** Error metrics for real space and Fourier space data, for the case where the lower (“reference”) half of the crystal is assumed to be imperfect.

Types of imperfection	Maximum phase (rad) due to parabolic displacement field	Minimum amplitude due to spherical inclusions	*Normalised RMS error* (*d_ph*) *for reconstructed phase*	*Normalised absolute error* (*r_ph*) *for reconstructed phase*	*Normalised RMS error* (*d_amp*) *for reconstructed amplitude*	*Normalised absolute error* (*r_amp*) *for reconstructed amplitude*	*Error metric* (*χ* ^2^) *in Fourier space*
original model for a displacement field throughout the crystal (ideal bottom half of the crystal)	0.25	0	0.1	0.2	0.6	0.1	1 × 10^−5^
displacement field exists in the entire crystal (bottom half of the crystal is deformed)	0.25	0	0.7	0.6	2	0.2	3 × 10^−3^
displacement field throughout the crystal and a slice 1pix(X) × 16pix(Z) × 64pix(Y) is removed from the bottom part of the crystal	0.25	0	0.7	0.6	4	0.2	3 × 10^−3^
displacement field throughout the crystal and a slice 32pix(X) × 1pix(Z) × 64pix(Y) is removed from the bottom part of the crystal	0.25	0	0.8	0.6	5	0.3	3 × 10^−3^

Maximum intensity is 10^9^ photons per voxel. The brightest voxel, corresponding to the origin of Fourier space, was excluded from the noise adding procedure as the appropriate intensity is multiplied by zero (see equation (11)) during the reconstruction process. Successive rows consider an ideal lower half of the crystal (as assumed by our reconstruction algorithm), followed by three different forms of departure from this assumed ideality. Weak displacement field and strong spherical inclusions are assumed throughout. For each case, five different error metrics (specified in equations (16), (17) and (18) of the main text) are given.

The *d* and *r* error metrics (see Table 1) for low SNR show that the phase reconstructions are more robust than the amplitude reconstructions for noisy data. It should be also noted that without noise, the phase reconstructions using the phase derivatives (equations (13–15)) produce the same level of errors as for direct phase reconstruction (equation (11)) for a smaller phase. The errors in the reconstruction of the amplitude are smaller if there is a larger difference between the scattering factor of the spherical inclusion and surrounding material.

**Figure 8:**
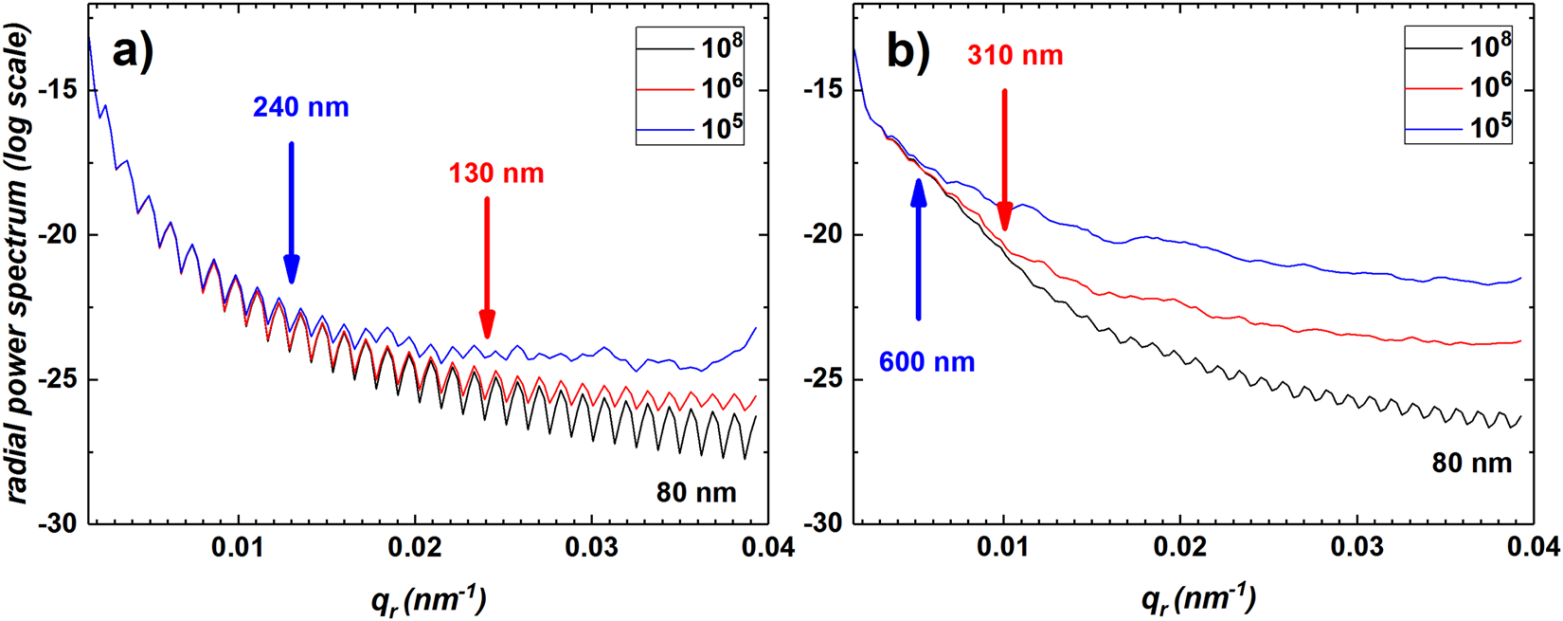
Investigation of the resolution of the simulated reconstructions obtained using our method. Radial power spectra of the intensity simulated using values for the phase and amplitude reconstructed from the intensity (with maximum intensity of 10^8^ (black lines), 10^6^ (red lines) and 10^5^ (blue lines)) simulated using the original model for the phase and amplitude. (**a**) The coefficient *γ* is chosen to yield a maximum phase shift of 0.25π. ${\beta }_{h}^{p}=0.9$ for spherical inclusions. (**b**) The coefficient *γ* is chosen to yield a maximum phase shift of 3π. ${\beta }_{h}^{p}=0$ for spherical inclusions. The achieved resolution is shown in colours corresponding to the initial maximum intensity. We chose the largest spatial frequencies, above which the power spectrum deviates significantly from that obtained using the essentially perfect reconstruction obtained at the highest photon flux, namely 80 nm, as indicators of the achieved resolution.

It should be noted that an 80 nm resolution was achieved only for a low level of noise as demonstrated in Fig. 8, which shows how the radial power spectrum depends on the maximum intensity level for two models shown in Figs 3 and 5. Note that this radial power spectrum is calculated using the synthesized three-dimensional diffracted intensity corresponding to each reconstruction at each specified maximum number of photons per voxel. We chose, as indicators of the achieved resolution, the points, where the radial power spectrum deviates significantly from that obtained using the essentially perfect reconstruction obtained at the highest flux. This highest-flux-data reconstruction, namely the reconstruction corresponding to 10^8^ photons per voxel in the most intensely illuminated voxel, was considered to have a reconstruction resolution equal to the real-space pixel size, namely 80 nm (see black curve in Fig. 8).

We close this section with some remarks on the sampling of the diffracted intensity data. While the simulations of this section have assumed, for simplicity, that these data are sampled along a Cartesian grid, in an actual experiment the detection frame of points will be different to the Cartesian grid used in the simulations. Interpolation will therefore be required – as is usual for BCDI experiments^51^ – e.g. with the transformations given in ref. 51 (see also refs 52 and 53). Importantly, whether these interpolations are linear or non-linear in terms of coordinates, the said interpolations will be linear in terms of detected intensities. Given that (i) the sum of Gaussian random processes is itself Gaussian, and (ii) all photon fluxes considered in the present paper are sufficiently high for Gaussian statistics to apply, the interpolation of the intensity data will (iii) lead to negligible artefacts provided that the detection frame has a sufficiently high resolution as specified by the rocking-curve sampling condition given by equation 4 in ref. 53 and (iv) preserve the Gaussian nature of the photon statistics, which will in turn leave unaltered the noise propagation properties of our algorithm as presented in the above numerical simulations. Some authors (see e.g., ref. 54) apply the iterative methods of BCDI for the intensity data set directly in the detection frame of points to preserve counting statistics. However, a more detailed consideration of the effects of interpolation of the intensity data, from the detection frame to a Cartesian frame, is beyond the scope of this paper and would form an interesting point of departure for future investigations.

## Discussion

Our means of deterministic coherent diffractive imaging, based on (11) and (12), may be viewed as a somewhat distant variant of Fourier holography (cf. a similar observation made in a different but related context in ref. 36). When the simple closed-form reconstruction of (11) and (12) is viewed from such a perspective, the far-field intensity data in the vicinity of a given Bragg peak, may be viewed as a generalised Fourier hologram in which the reference wave is produced by the locally-cylindrical Young–type boundary waves^55^ scattered by the edges of the crystal.

The above physical picture, for how our reconstruction method works, has the following corresponding loose mathematical picture which may be taken as giving some guidance to the detailed calculations presented in the main text and Supplementary Section of our paper. Loosely, then, our infinite-extent crystal structure is truncated by a rectangular prism envelope given by a product of top-hat (“rect”) functions in x, y and z, before being Fourier transformed, and the squared magnitude taken, to yield the raw intensity diffraction data for our method. Multiplying by q_x_·q_y_·q_z_ and then inverse Fourier transforming (equation (11)), will - by recalling both the Fourier derivative and convolution theorems of Fourier analysis - yield the derivative of the autocorrelation of the desired structure. The derivative of the correlation of two functions is equal to the correlation of one function with the derivative of the other; the derivative of the top-hat function yields spatially separated Dirac deltas which then sift out (via the sifting property of the Dirac delta) the spatially separated independent reconstructions (“B” and “C” terms in equation (12)).

It is self-evident that the solution provided by (11) and (12) is the solution to an inverse problem which is well-posed in the sense of Hadamard^56^. The absolute simplicity of the coherent diffractive imaging reconstruction process is obtained at the price of the stated, rather strong, simplifying assumptions (rectangular crystal whose imperfections are restricted to any half of the scattering volume, kinematical scattering etc.). Regarding these strong assumptions, we make two points:

(i) One can always determine *a posteriori* whether or not the stated assumptions regarding imperfections filling no more than half of the crystalline volume have been met, by simply inspecting whether or not there are overlapping regions in the multiple reconstructions furnished by the auxiliary function *U* given by (11).
(ii) A key motivation of the present work is to suggest and hopefully stimulate a relatively new line of research in the coherent diffractive imaging community, in pursuing closed-form solutions to the problem of phasing Fourier-modulus data. Our approach may be counterpointed against the much more prevalent iterative approaches, which have already been brought to a high stage of perfection, but which suffer from the lack of conceptual clarity which closed-form solutions provide. We (S.G. Podorov, K.M. Pavlov & D.M. Paganin) have previously expressed such a motivation for deterministic coherent diffractive imaging in our previous works in a two-dimensional context^36, 57^, which have stimulated some further work in the field (e.g., refs 44, 45, 58–66 and many others). It is our hope that the present paper, together with refs 67 and 68, may stimulate further work in three-dimensional deterministic CDI.

We close this discussion with some obvious extensions of the work presented here: (i) Bragg CDI has resolutions larger than typical unit cells, and thus is not affected by the symmetry of the unit cell. Hence the inversion procedure presented in our article is valid for any crystal which can be described as repeated unit cells, irrespective of the crystal space group^69^; (ii) Another obvious extension is to the case of general convex polyhedra (convex faceted crystals); (iii) The deterministic reconstruction method of the present paper may without modification be applied to “imperfections” which lie partly within and partly outside, or even entirely outside, the crystal (i.e., ideal and non-ideal parts can form one piece or be separated) (cf. refs 36, 58, 68); (iv) The deterministic reconstruction method of the present paper may subsequently be used to seed an iterative refinement, for structures which weakly violate the validity conditions for the deterministic algorithm (cf. ref. 36); (v) The methods of compressed sensing (see e.g. refs 70, 71) may be fruitfully applied to the question of reconstructing the complex polarisability for defects that are distributed throughout the entire volume of an otherwise perfect crystal but which are either suitably sparse or may be rendered so through an appropriate sparsifying operator; (vi) It would be interesting to systematically extend the preliminary studies given above regarding the deviations from ideality of the part of the crystal which we assume to be known; (vii) The methods developed in this paper might be adapted to give a technique for deterministic protein crystallography, e.g. by modifying the well-known iterative techniques^72^ along the following or similar lines: a small crystal of an unknown protein is attached to a known crystalline reference structure, of similar size, and with very similar lattice constants.

## Conclusions

In summary, we introduced a deterministic Bragg Coherent Diffraction Imaging approach, which was successfully employed to analytically reconstruct the amplitude (structure factor) and phase (displacement field) from noisy simulated 3D diffracted intensity distributions. This robust unambiguous reconstruction algorithm uses a holographical type interference between “ideal” and “damaged” part of crystalline structure, where the “damaged” part is less than a half of the total volume of the object. The approach can be used for both weak and strong phase objects. The algorithm has been tested using a variety of simulated scenarios, performing well in the presence of both (i) realistic levels of noise, and (ii) departures from ideality in the part of the crystal that is assumed to be undamaged.

## Electronic supplementary material

Supplementary Movie 2

Supplementary Movie 3

Supplementary Movie 4

Supplementary Movie 5

Supplementary Movie 7

Supplementary Section
