# Supplementary material for: Deterministic Bragg Coherent Diffraction Imaging
Source: Sci Rep. 2017 Apr 25;7:1132. doi: 10.1038/s41598-017-01164-x (PMC5430781; doi:10.1038/s41598-017-01164-x)
Supplement: Supplementary file 6 — Supplementary Section [file 41598_2017_1164_MOESM6_ESM.pdf]

Deterministic Bragg Coherent Diffraction Imaging

Konstantin M. Pavlov[1),2)*], Vasily I. Punegov[3),4)], Kaye S. Morgan[2),5)], Gerd Schmalz[1)] and David M. Paganin[2)]

[1]School of Science and Technology, University of New England, NSW 2351, Australia

[2]School of Physics and Astronomy, Monash University, VIC 3800, Australia

[3]Komi Research Center, Ural Division, Russian Academy of Sciences, Syktyvkar, 167982, Russian Federation

[4]Syktyvkar State University, Syktyvkar, 167001, Russian Federation

[5]Institute for Advanced Studies and Chair of Biomedical Physics, Technische Universität München, Bayern, 85748, Germany

*Correspondence e-mail: kpavlov@une.edu.au

We begin with equations (11) and (12) from the main text:

$$
\begin{aligned}
U(\mathbf{R}=(x,y,z)) &= |B_e|^{-2}\left|\chi_h^{id}\right|^{-2}\frac{1}{(2\pi)^{3/2}}\iiint q_x q_y q_z \hat{I}_{kin}\exp(iq_x x+iq_y y+iq_z z)dq_x dq_y dq_z \\
&= A+\sum_{j=1}^{8}(B_j+C_j)+D.
\end{aligned}
\tag{S1}
$$

Explicit expressions for the terms A, B_j, C_j and D are calculated below.

$$A(\mathbf{R})=\frac{N_x^2N_y^2N_z^2}{(2\pi)^{3/2}}\iiint q_xq_yq_z\left\{\frac{\sin(q_xaN_x/2)}{q_xaN_x/2}\frac{\sin(q_yaN_y/2)}{q_yaN_y/2}\frac{\sin(q_zaN_z/2)}{q_zaN_z/2}\right\}^2 e^{iq_xx+iq_yy+iq_zz}dq_xdq_ydq_z$$

$$=\frac{iN_x^2N_y^2N_z^2}{(2\pi)^{3/2}}\partial_{xyz}^3\iiint\left\{\frac{\sin(q_xaN_x/2)}{q_xaN_x/2}\frac{\sin(q_yaN_y/2)}{q_yaN_y/2}\frac{\sin(q_zaN_z/2)}{q_zaN_z/2}\right\}^2 e^{iq_xx+iq_yy+iq_zz}dq_xdq_ydq_z$$

$$=\left|q_x=2\pi f_x;\ q_y=2\pi f_y;\ q_z=2\pi f_z\right|=$$

$$=\frac{i8a^3\pi^3N_x^2N_y^2N_z^2}{a^3(2\pi)^{3/2}}\partial_{xyz}^3\iiint\left\{\frac{\sin(\pi f_xaN_x)}{\pi f_xaN_x}\frac{\sin(\pi f_yaN_y)}{\pi f_yaN_y}\frac{\sin(\pi f_zaN_z)}{\pi f_zaN_z}\right\}^2 e^{i2\pi(f_xx+f_yy+f_zz)}df_xdf_ydf_z$$

$$=\frac{i8\pi^3N_xN_yN_z}{a^3(2\pi)^{3/2}}\partial_{xyz}^3\Lambda\left(\frac{x}{aN_x}\right)\Lambda\left(\frac{y}{aN_y}\right)\Lambda\left(\frac{z}{aN_z}\right)$$

$$=\frac{-i(2\pi)^{3/2}\operatorname{sgn}(x)\operatorname{sgn}(y)\operatorname{sgn}(z)}{a^6}$$

$$\times[\theta(x+aN_x)-\theta(x-aN_x)][\theta(y+aN_y)-\theta(y-aN_y)][\theta(z+aN_z)-\theta(z-aN_z)]. \tag{S2}$$

Here, sgn(x) denotes the signum function, $\Lambda(x)$ denotes the triangle function and $\theta(x)$ denotes the Heaviside step function. We used a tabulated integral (see e.g., p. 14 in ref. 73).

Evidently, A is the term proportional to the shape function of the sample.

The terms, $B_{1,2,3,4,5,6,7,8}$ and $C_{1,2,3,4,5,6,7,8}$ are straight-forward results of the Fourier shift theorem (see e.g., p.8 in ref. 73). Explicitly, we have:

$$B_1(\mathbf{R})=\frac{i}{a^3(2\pi)^{3/2}}$$

$$\times\iiint\left\{Z(\mathbf{q})\sum_{p'=1}^{N}e^{-i\mathbf{q}\cdot\mathbf{R}_{p'}}\left[\left(\beta_h^{p'}\right)e^{-i\mathbf{h}\cdot\mathbf{u}_{p'}}-1\right]\right\}e^{iq_x\left(x+a\left(N_x-\frac{1}{2}\right)\right)}e^{iq_y\left(y+a\left(N_y-\frac{1}{2}\right)\right)}e^{iq_z\left(z+a\left(N_z-\frac{1}{2}\right)\right)}dq_xdq_ydq_z$$

$$=i\frac{(2\pi)^{3/2}}{a^6}\left[\begin{array}{l}\left(\beta_h\left(x+a\left(N_x-\frac{1}{2}\right),y+a\left(N_y-\frac{1}{2}\right),z+a\left(N_z-\frac{1}{2}\right)\right)\right)\\ \times e^{-i\mathbf{h}\cdot\mathbf{u}\left(x+a\left(N_x-\frac{1}{2}\right),y+a\left(N_y-\frac{1}{2}\right),z+a\left(N_z-\frac{1}{2}\right)\right)}-1\end{array}\right] \tag{S3}$$

$$B_2(\mathbf{R})=-i\frac{(2\pi)^{3/2}}{a^6}\left[\begin{array}{l}\left(\beta_h\left(x-\frac{a}{2},y+a\left(N_y-\frac{1}{2}\right),z+a\left(N_z-\frac{1}{2}\right)\right)\right)\\ \times e^{-i\mathbf{h}\cdot\mathbf{u}\left(x-\frac{a}{2},y+a\left(N_y-\frac{1}{2}\right),z+a\left(N_z-\frac{1}{2}\right)\right)}-1\end{array}\right] \tag{S4}$$

$$B_3(\mathbf{R}) = -i\frac{(2\pi)^{3/2}}{a^6}\left[\begin{array}{l}\left(\beta_h\left(x+a\left(N_y-\frac{1}{2}\right), y-\frac{a}{2}, z+a\left(N_z-\frac{1}{2}\right)\right)\right) \\ \times e^{-i\mathbf{h}\cdot\mathbf{u}\left(x+a\left(N_x-\frac{1}{2}\right), y-\frac{a}{2}, z+a\left(N_z-\frac{1}{2}\right)\right)} -1\end{array}\right] \tag{S5}$$

$$B_4(\mathbf{R}) = i\frac{(2\pi)^{3/2}}{a^6}\left[\begin{array}{l}\left(\beta_h\left(x-\frac{a}{2}, y-\frac{a}{2}, z+a\left(N_z-\frac{1}{2}\right)\right)\right) \\ \times e^{-i\mathbf{h}\cdot\mathbf{u}\left(x-\frac{a}{2}, y-\frac{a}{2}, z+a\left(N_z-\frac{1}{2}\right)\right)} -1\end{array}\right] \tag{S6}$$

$$B_5(\mathbf{R}) = -i\frac{(2\pi)^{3/2}}{a^6}\left[\begin{array}{l}\left(\beta_h\left(x+a\left(N_x-\frac{1}{2}\right), y+a\left(N_y-\frac{1}{2}\right), z-\frac{a}{2}\right)\right) \\ \times e^{-i\mathbf{h}\cdot\mathbf{u}\left(x+a\left(N_x-\frac{1}{2}\right), y+a\left(N_y-\frac{1}{2}\right), z-\frac{a}{2}\right)} -1\end{array}\right] \tag{S7}$$

$$B_6(\mathbf{R}) = i\frac{(2\pi)^{3/2}}{a^6}\left[\begin{array}{l}\left(\beta_h\left(x-\frac{a}{2}, y+a\left(N_y-\frac{1}{2}\right), z-\frac{a}{2}\right)\right) \\ \times e^{-i\mathbf{h}\cdot\mathbf{u}\left(x-\frac{a}{2}, y+a\left(N_y-\frac{1}{2}\right), z-\frac{a}{2}\right)} -1\end{array}\right] \tag{S8}$$

$$B_7(\mathbf{R}) = i\frac{(2\pi)^{3/2}}{a^6}\left[\begin{array}{l}\left(\beta_h\left(x+a\left(N_x-\frac{1}{2}\right), y-\frac{a}{2}, z-\frac{a}{2}\right)\right) \\ \times e^{-i\mathbf{h}\cdot\mathbf{u}\left(x+a\left(N_x-\frac{1}{2}\right), y-\frac{a}{2}, z-\frac{a}{2}\right)} -1\end{array}\right] \tag{S9}$$

$$B_8(\mathbf{R}) = -i\frac{(2\pi)^{3/2}}{a^6}\left[\left(\beta_h\left(x-\frac{a}{2}, y-\frac{a}{2}, z-\frac{a}{2}\right)\right)e^{-i\mathbf{h}\cdot\mathbf{u}\left(x-\frac{a}{2}, y-\frac{a}{2}, z-\frac{a}{2}\right)} -1\right] \tag{S10}$$

$$\begin{aligned} C_1(\mathbf{R}) &= \frac{i}{a^3(2\pi)^{3/2}} \\ &\times\iiint\left\{\sum_{p'=1}^{N} e^{i\mathbf{q}\cdot\mathbf{R}_{p'}}\left[\left(\beta_h^{p'}\right)^* e^{i\mathbf{h}\cdot\mathbf{u}_{p'}} -1\right]\right\} e^{iq_x\left(x+\frac{a}{2}\right)} e^{iq_y\left(y+\frac{a}{2}\right)} e^{iq_z\left(z+\frac{a}{2}\right)} dq_x dq_y dq_z \\ &= i\frac{(2\pi)^{3/2}}{a^6}\left[\left(\beta_h^*\left(-x-\frac{a}{2}, -y-\frac{a}{2}, -z-\frac{a}{2}\right)\right)e^{i\mathbf{h}\cdot\mathbf{u}\left(-x-\frac{a}{2}, -y-\frac{a}{2}, -z-\frac{a}{2}\right)} -1\right]\end{aligned} \tag{S11}$$

$$C_2(\mathbf{R}) = -i\frac{(2\pi)^{3/2}}{a^6}\left[\begin{array}{l}\left(\beta_h^*\left(-x-\frac{a}{2}+aN_x, -y-\frac{a}{2}, -z-\frac{a}{2}\right)\right) \\ \times e^{i\mathbf{h}\cdot\mathbf{u}\left(-x-\frac{a}{2}+aN_x, -y-\frac{a}{2}, -z-\frac{a}{2}\right)} -1\end{array}\right] \tag{S12}$$

$$C_3(\mathbf{R}) = -i\frac{(2\pi)^{3/2}}{a^6}\left[\begin{array}{l}\left(\beta_h^*\left(-x-\frac{a}{2},-y-\frac{a}{2}+aN_y,-z-\frac{a}{2}\right)\right)\\ \times e^{i\mathbf{h}\cdot\mathbf{u}\left(-x-\frac{a}{2},-y-\frac{a}{2}+aN_y,-z-\frac{a}{2}\right)}-1\end{array}\right] \quad \text{(S13)}$$

$$C_4(\mathbf{R}) = i\frac{(2\pi)^{3/2}}{a^6}\left[\begin{array}{l}\left(\beta_h^*\left(-x-\frac{a}{2}+aN_x,-y-\frac{a}{2}+aN_y,-z-\frac{a}{2}\right)\right)\\ \times e^{i\mathbf{h}\cdot\mathbf{u}\left(-x-\frac{a}{2}+aN_x,-y-\frac{a}{2}+aN_y,-z-\frac{a}{2}\right)}-1\end{array}\right] \quad \text{(S14)}$$

$$C_5(\mathbf{R}) = -i\frac{(2\pi)^{3/2}}{a^6}\left[\begin{array}{l}\left(\beta_h^*\left(-x-\frac{a}{2},-y-\frac{a}{2},-z-\frac{a}{2}+aN_z\right)\right)\\ \times e^{i\mathbf{h}\cdot\mathbf{u}\left(-x-\frac{a}{2},-y-\frac{a}{2},-z-\frac{a}{2}+aN_z\right)}-1\end{array}\right] \quad \text{(S15)}$$

$$C_6(\mathbf{R}) = i\frac{(2\pi)^{3/2}}{a^6}\left[\begin{array}{l}\left(\beta_h^*\left(-x-\frac{a}{2}+aN_x,-y-\frac{a}{2},-z-\frac{a}{2}+aN_z\right)\right)\\ \times e^{i\mathbf{h}\cdot\mathbf{u}\left(-x-\frac{a}{2}+aN_x,-y-\frac{a}{2},-z-\frac{a}{2}+aN_z\right)}-1\end{array}\right] \quad \text{(S16)}$$

$$C_7(\mathbf{R}) = i\frac{(2\pi)^{3/2}}{a^6}\left[\begin{array}{l}\left(\beta_h^*\left(-x-\frac{a}{2},-y-\frac{a}{2}+aN_y,-z-\frac{a}{2}+aN_z\right)\right)\\ \times e^{i\mathbf{h}\cdot\mathbf{u}\left(-x-\frac{a}{2},-y-\frac{a}{2}+aN_y,-z-\frac{a}{2}+aN_z\right)}-1\end{array}\right] \quad \text{(S17)}$$

$$C_8(\mathbf{R}) = -i\frac{(2\pi)^{3/2}}{a^6}\left[\begin{array}{l}\left(\beta_h^*\left(-x-\frac{a}{2}+aN_x,-y-\frac{a}{2}+aN_y,-z-\frac{a}{2}+aN_z\right)\right)\\ \times e^{i\mathbf{h}\cdot\mathbf{u}\left(-x-\frac{a}{2}+aN_x,-y-\frac{a}{2}+aN_y,-z-\frac{a}{2}+aN_z\right)}-1\end{array}\right] \quad \text{(S18)}$$

Clearly, this gives a series of 16 independent reconstructions of the desired complex field $\beta_h e^{-i\mathbf{h}\cdot\mathbf{u}}$, and its complex conjugate. Conditions for these reconstructions to not overlap, with any of the other terms in the auxiliary function *U*, are discussed in the main text.

We close the supplementary section with a calculation of the *D* function:

$$
\begin{aligned}
D(\mathbf{R}) &= \frac{1}{(2\pi)^{3/2}} \iiint q_x q_y q_z \left| Z(\mathbf{q}) \sum_{p'=1}^{N} \exp\left(-i\mathbf{q}\cdot\mathbf{R}_{p'}\right) \left[\beta_h^p \exp\left(-i\mathbf{h}\cdot\mathbf{u}_{p'}\right) - 1\right] \right|^2 \exp(i\mathbf{q}\cdot\mathbf{R}) dq_x dq_y dq_z \\
&= \frac{i}{a^6 (2\pi)^{3/2}} \partial^3_{xyz} \iiint \left| \iiint d\mathbf{r}' e^{-i\mathbf{q}\cdot\mathbf{r}'} \left[\beta_h(\mathbf{r}') e^{-i\mathbf{h}\cdot\mathbf{u}(\mathbf{r}')} - 1\right] \right|^2 \exp(i\mathbf{q}\cdot\mathbf{R}) dq_x dq_y dq_z \\
&= \frac{i}{a^6 (2\pi)^{3/2}} \partial^3_{xyz} \iiint \left\{ \iiint d\mathbf{r}' e^{-i\mathbf{q}\cdot\mathbf{r}'} \left[\beta_h(\mathbf{r}') e^{-i\mathbf{h}\cdot\mathbf{u}(\mathbf{r}')} - 1\right] \right\} \left\{ \iiint d\mathbf{r}'' e^{i\mathbf{q}\cdot\mathbf{r}''} \left[\beta_h^*(\mathbf{r}'') e^{i\mathbf{h}\cdot\mathbf{u}(\mathbf{r}'')} - 1\right] \right\} e^{i\mathbf{q}\cdot\mathbf{R}} dq_x dq_y dq_z \\
&= \frac{i}{a^6 (2\pi)^{3/2}} \partial^3_{xyz} \left\{ \iiint d\mathbf{r}' \iiint d\mathbf{r}'' \left[\beta_h(\mathbf{r}') e^{-i\mathbf{h}\cdot\mathbf{u}(\mathbf{r}')} - 1\right] \left[\beta_h^*(\mathbf{r}'') e^{i\mathbf{h}\cdot\mathbf{u}(\mathbf{r}'')} - 1\right] \iiint e^{i\mathbf{q}\cdot(-\mathbf{r}'+\mathbf{r}''+\mathbf{R})} dq_x dq_y dq_z \right\} \\
&= \frac{i(2\pi)^{3/2}}{a^6} \partial^3_{xyz} \left\{ \iiint d\mathbf{r}' \iiint d\mathbf{r}'' \left[\beta_h(\mathbf{r}') e^{-i\mathbf{h}\cdot\mathbf{u}(\mathbf{r}')} - 1\right] \left[\beta_h^*(\mathbf{r}'') e^{i\mathbf{h}\cdot\mathbf{u}(\mathbf{r}'')} - 1\right] \delta(-\mathbf{r}' + \mathbf{r}'' + \mathbf{R}) \right\} \\
&= \frac{i(2\pi)^{3/2}}{a^6} \partial^3_{xyz} \left\{ \iiint d\mathbf{r}' \left[\beta_h(\mathbf{r}') e^{-i\mathbf{h}\cdot\mathbf{u}(\mathbf{r}')} - 1\right] \left[\beta_h^*(\mathbf{r}' - \mathbf{R}) e^{i\mathbf{h}\cdot\mathbf{u}(\mathbf{r}'-\mathbf{R})} - 1\right] \right\}.
\end{aligned}
\qquad \text{(S19)}
$$

We see that D is the third-order derivative of the cross-correlation function, which will be located in the central part of the auxiliary function U.

Note that, in the above calculations, we have used the following connection between a discrete sum and a continuous integral:

$$
\sum_{j=0}^{N-1} T(j) \exp(iqaj) = \frac{-iqa}{1-\exp(iqa)} \int_0^{Na} \exp(iqx) \frac{T(x)}{a} dx, \qquad \text{(S20)}
$$

where $T(x)$ is a step-like function. That is, $T(x)$ is constant over each step of length a.

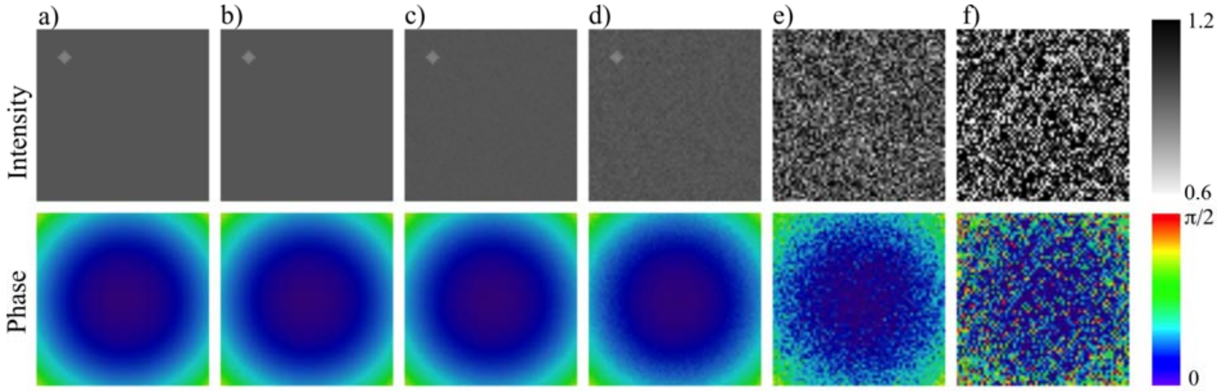

Supplementary Figure 3. The amplitude (shown in grey scale) and phase (shown in colour scale) for a 2D slice going through the innermost sphere. The coefficient γ is chosen to yield a maximum phase shift of 0.25π. $\beta_h^p = 0.9$ for spherical inclusions. (a) – the original model, (b) – the reconstruction in absence of noise, (c) – the reconstruction with maximum intensity of 10^9 photons per voxel, (d) – the reconstruction with maximum intensity of 10^8 photons per voxel, (e) – the reconstruction with maximum intensity of 10^6 photons per voxel, (f) – the reconstruction with maximum intensity of 10^5 photons per voxel.

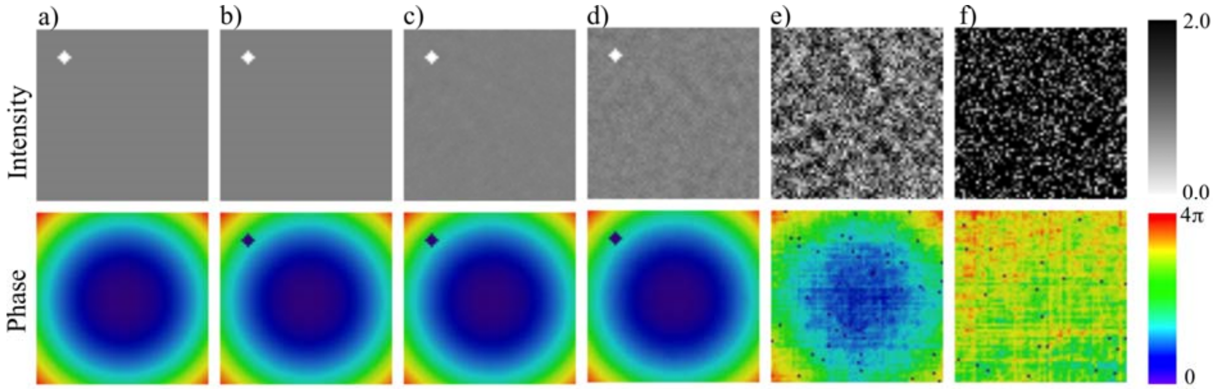

Supplementary Figure 5. The amplitude (shown in grey scale) and phase (shown in colour scale) for a 2D slice going through the most inner sphere. The coefficient γ is chosen to yield a maximum phase shift of 3π. $\beta_h^p = 0$ for spherical inclusions. (a) – the original model, (b) – the reconstruction in absence of noise, (c) – the reconstruction with maximum intensity of 10^9, (d) – the reconstruction with maximum intensity of 10^8, (e) – the reconstruction with maximum intensity of 10^6, (f) – the reconstruction with maximum intensity of 10^5.

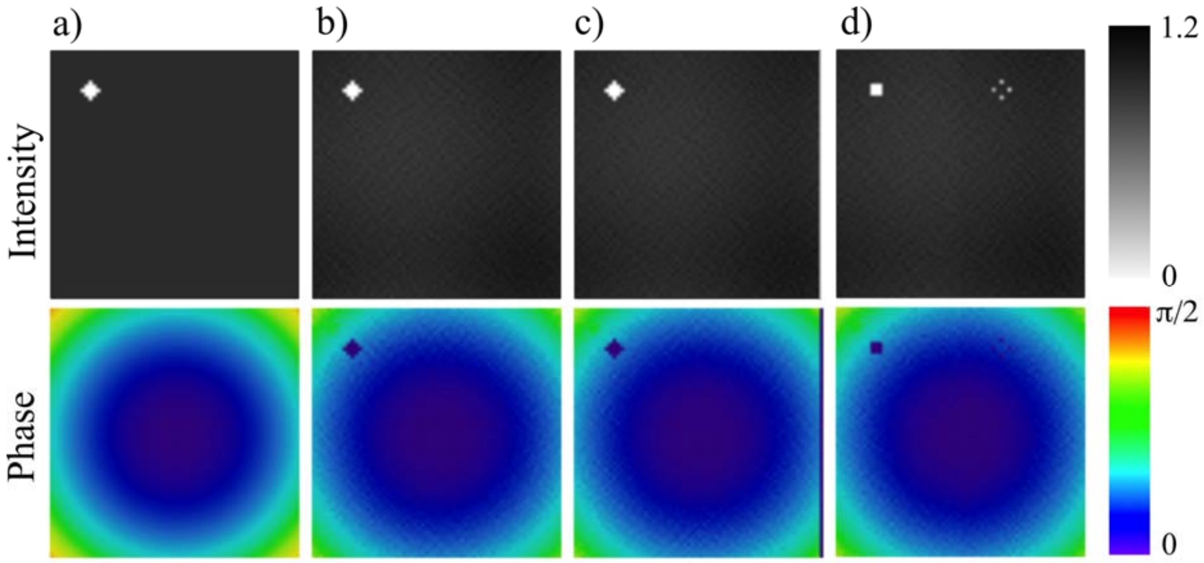

Supplementary Figure 7. The amplitude (shown in grey scale) and phase (shown in colour scale) for a 2D slice going through the most inner sphere. The coefficient γ is chosen to yield a maximum phase shift of 0.25π. $\beta_h^p = 0$ for spherical inclusions. The maximum intensity is 10^9 photons per voxel. (a) – the original model for a displacement field throughout the crystal (shown only the upper part of the crystal), (b) – the reconstruction for a displacement field throughout the crystal, (c) – the reconstruction for a displacement field throughout the crystal when a slice 1pixel(X)×16pixels(Z)×64pixels(Y) is removed from the bottom part of the crystal, (d) – the reconstruction for a displacement field throughout the crystal when a slice 32pixels(X)×1pixel(Z)×64pixels(Y) is removed from the bottom part of the crystal.

Supplementary Movie captions:

Supplementary Movie 2. 3D distributions of diffracted intensity shown on a logarithmic scale. The coefficient γ is chosen to yield a maximum phase shift of 0.25π. $\beta_h^p = 0.9$ for spherical inclusions. (a), (c), (e) and (g) - the intensity simulated using the original model for the phase and amplitude; (b), (d), (f) and (h) the intensity simulated using the reconstructed values for the phase and amplitude; (a) and (b) – no noise; (c) and (d) – maximum intensity of 10^9 photons per voxel; (e) and (f) – maximum intensity of 10^8 photons per voxel; (g) and (h) – maximum intensity of 10^5 photons per voxel. In all panels, Fourier-space coordinates q_x, q_y and q_z lie within the range $\pm 1.96 \times 10^{-2}$ nm^{-1}.

Supplementary Movie 3. The amplitude (shown in grey scale) and phase (shown in colour scale). The coefficient γ is chosen to yield a maximum phase shift of 0.25π. $\beta_h^p = 0.9$ for spherical inclusions. (a) – the original model, (b) – the reconstruction in absence of noise, (c) – the reconstruction with maximum intensity of 10^9 photons per voxel, (d) – the reconstruction with maximum intensity of 10^8 photons per voxel, (e) – the reconstruction with maximum intensity of 10^6 photons per voxel, (f) – the reconstruction with maximum intensity of 10^5 photons per voxel.

Supplementary Movie 4. 3D distributions of diffracted intensity shown on a logarithmic scale. The coefficient γ is chosen to yield a maximum phase shift of 3π. $\beta_h^p = 0$ for spherical inclusions. (a), (c), (e) and (g) - the intensity simulated using the original model for the phase and amplitude; (b), (d), (f) and (h) the intensity simulated using the reconstructed values for the phase and amplitude; (a) and (b) – no noise; (c) and (d) – maximum intensity of 10^9; (c) and (d) – maximum intensity of 10^8; (c) and (d) – maximum intensity of 10^5. In all panels, Fourier-space coordinates q_x, q_y and q_z lie within the range $\pm 1.96 \times 10^{-2}$ nm^{-1}.

Supplementary Movie 5. The amplitude (shown in grey scale) and phase (shown in colour scale). The coefficient γ is chosen to yield a maximum phase shift of 3π. $\beta_h^p = 0$ for spherical inclusions. (a) – the original model, (b) – the reconstruction in absence of noise, (c) – the reconstruction with maximum intensity of 10^9, (d) – the reconstruction with maximum intensity of 10^8, (e) – the reconstruction with maximum intensity of 10^6, (f) – the reconstruction with maximum intensity of 10^5.

Supplementary Movie 7. The amplitude (shown in grey scale) and phase (shown in colour scale). The coefficient γ is chosen to yield a maximum phase shift of 0.25π. $\beta_h^p = 0$ for spherical inclusions. The maximum intensity is 10^9 photons per voxel. (a) – the original model for a displacement field throughout the crystal (shown only the upper part of the crystal), (b) – the reconstruction for a displacement field throughout the crystal, (c) – the reconstruction for a displacement field throughout the crystal when a slice 1pixel(X)×16pixels(Z)×64pixels(Y) is removed from the bottom part of the crystal, (d) – the reconstruction for a displacement field throughout the crystal when a slice 32pixels(X)×1pixel(Z)×64pixels(Y) is removed from the bottom part of the crystal.
